# Flavonoids in atopic dermatitis: mechanisms, delivery innovations, and translational strategies

**DOI:** 10.3389/fphar.2025.1631977

**Published:** 2025-08-22

**Authors:** Dongdong Li, Yusheng Han, Jingjing Zhou, Jing Chen, Hong Liang Tey, Timothy T. Y. Tan

**Affiliations:** ^1^ College of Basic Medical Sciences, Heilongjiang University of Chinese Medicine, Harbin, China; ^2^ School of Chemistry, Chemical Engineering and Biotechnology, Nanyang Technological University, Singapore, Singapore; ^3^ Experimental Teaching and Practical Training Center, Heilongjiang University of Chinese Medicine, Harbin, China; ^4^ Department of Dermatology, Chongqing Traditional Chinese Medicine Hospital, Chongqing, China; ^5^ National Skin Centre, Singapore, Singapore; ^6^ Skin Research Institute of Singapore, Singapore, Singapore; ^7^ Lee Kong Chian School of Medicine, Nanyang Technological University, Singapore, Singapore

**Keywords:** natural flavonoids, atopic dermatitis, botanical drugs, multi-target pharmacological actions, novel drug delivery systems

## Abstract

**Objective:**

To provide a comprehensive narrative synthesis of recent advances in the pharmacological actions and therapeutic potential of natural flavonoids in atopic dermatitis (AD), with emphasis on their multi-target pharmacological effects across core pathological mechanisms. The review also addresses pharmacokinetic limitations, formulation challenges, delivery innovations, safety concerns, and emerging clinical evidence to inform translational research and therapeutic development.

**Methods:**

This narrative review is based on a targeted literature search of PubMed, Web of Science, ScienceDirect, and SpringerLink, covering English-language, peer-reviewed articles published between 2010 and 2025. Search terms included natural flavonoid metabolites (e.g., quercetin, baicalin, epigallocatechin-3-gallate [EGCG]) combined using Boolean operators (e.g., AND, OR) with keywords related to atopic dermatitis, its underlying mechanisms, and therapeutic interventions. Studies focusing on *in vitro*, *in vivo*, or clinical evaluations of mechanistic pathways, therapeutic potential, or delivery strategies were included, while those addressing synthetic flavonoids, non-AD models, or lacking mechanistic relevance were excluded. This review does not follow a systematic review protocol.

**Results:**

Natural flavonoids exert multi-target effects in AD models by restoring skin barrier integrity, modulating immune and chemokine dysregulation, alleviating pruritus, regulating microbial homeostasis and programmed cell death, and attenuating oxidative stress. However, pharmacokinetic and physicochemical limitations such as poor solubility, low bioavailability, metabolic instability, and limited dermal targeting currently constrain clinical application. Potential safety concerns, including hepatotoxicity and endocrine disruption, also warrant careful evaluation. To address these challenges, advanced delivery platforms including microneedles, hydrogels, nanocarriers, microsponges, and liposomes have been explored to improve dermal delivery. Additionally, oral delivery systems developed in other inflammatory and oncological models provide valuable insights for guiding translational strategies in AD. Preliminary clinical evidence suggests potential benefits of flavonoid-based interventions; nevertheless, larger and well-controlled trials are necessary to substantiate their pharmacological effects and evaluate long-term safety.

**Conclusion:**

Natural flavonoids exhibit multi-target effects in AD by modulating core pathological processes. Although challenges such as limited bioavailability and safety concerns continue to impede clinical translation, these limitations may be addressed through the optimization of delivery strategies, rigorous pharmacokinetic and toxicological assessments, mechanism-driven *in vitro*, *in vivo*, *ex vivo* studies, and well-designed clinical trials.

## 1 Introduction

Atopic dermatitis (AD) is a complex chronic inflammatory skin disease driven by genetic predisposition (Loset et al., 2019), immune dysregulation (Kim et al., 2019), and environmental factors (Chong et al., 2022). Globally, the prevalence of AD reaches up to 20% in children and approximately 10% in adults (Hadi et al., 2021; Silverberg et al., 2021). Clinically, AD is characterized by intense pruritus and eczematous lesions, frequently accompanied by sleep disturbances and psychological comorbidities (Ariëns et al., 2019; Schmidt and de Guzman Strong, 2021; Casella et al., 2024). These symptoms substantially impair quality of life and impose a significant socioeconomic burden.

Currently, topical corticosteroids, calcineurin inhibitors, and antihistamines remain the mainstay treatments for AD; however, long-term use is associated with skin atrophy, immunosuppression, diminished efficacy, and a high risk of relapse (Broeders et al., 2016). Although biologic agents have improved outcomes for patients with moderate-to-severe AD, therapeutic responses remain heterogeneous, and many patients experience suboptimal long-term efficacy or intolerance (Bangert et al., 2021; David et al., 2023; Rothenberg-Lausell et al., 2024). Moreover, treatment costs, poor adherence, and psychological comorbidities continue to challenge sustained disease management (Guttman-Yassky et al., 2025).

Flavonoids are natural polyphenolic metabolites abundantly found in medicinal herbs, fruits, vegetables, and tea (Hussain et al., 2022). Owing to their antioxidant, anti-inflammatory, and immunomodulatory properties, flavonoids have attracted growing interest in pharmaceutical and biotherapeutic research (Dere and Khan, 2020). Recent studies have highlighted their promising potential in the prevention and treatment of AD (Wu et al., 2021), attributed to their multifaceted activities such as anti-inflammatory (Maleki et al., 2019), antioxidant (Fu K. et al., 2024), immunomodulatory (Zeinali et al., 2017; Ribeiro et al., 2018), and barrier-repairing effects (Park C.-H. et al., 2020).

**FIGURE 1:**
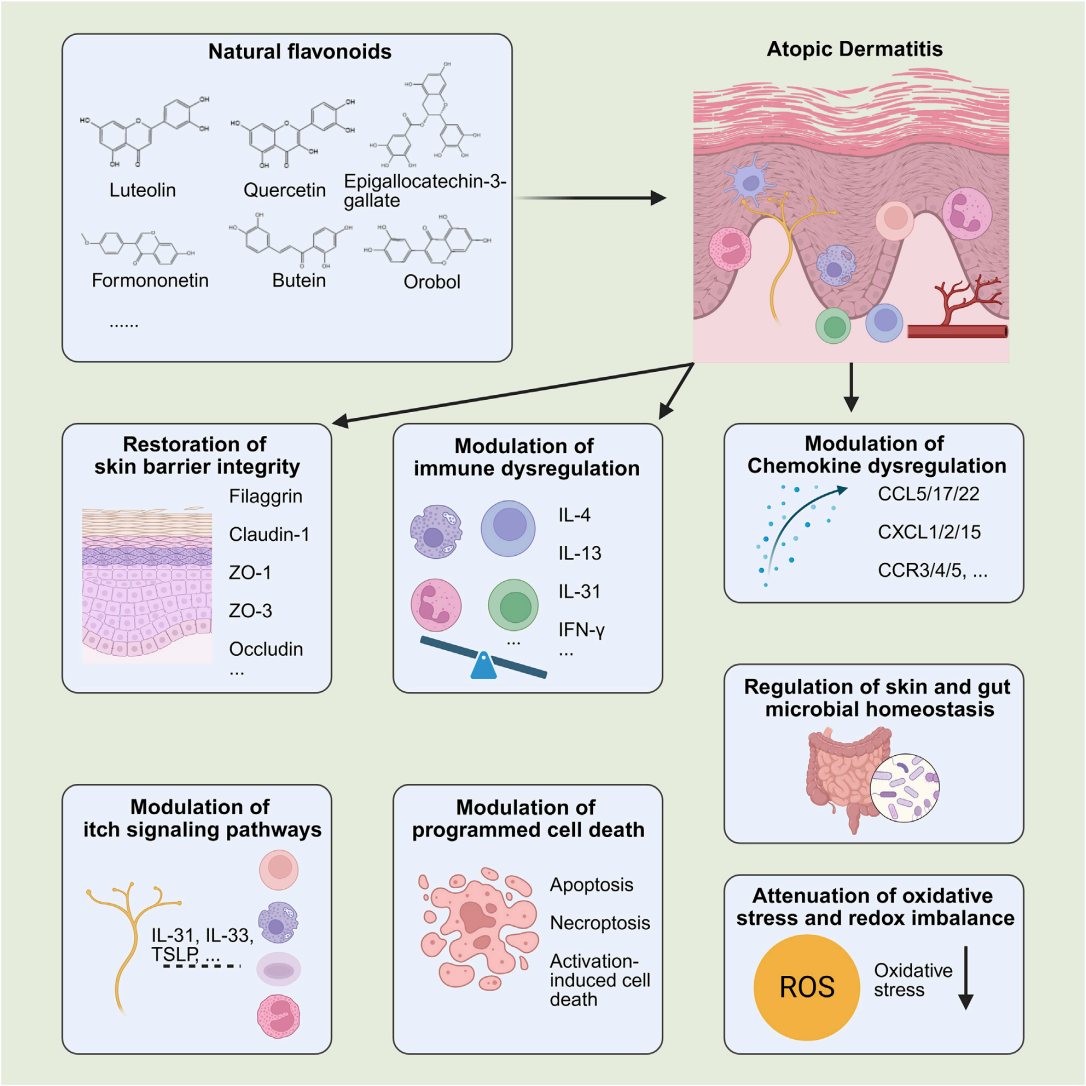
Pharmacological effects of flavonoids in AD. By BioRender. Note: ZO-, Zonula Occludens-; IL-, interleukin; TSLP, thymic stromal lymphopoietin; IFN-γ, interferon-gamma; CXCL, C-X-C motif chemokine ligand; CCL, C-C motif chemokine ligand; CCR-, C-C chemokine receptor type 3.

This review provides a comprehensive narrative overview of recent advances in flavonoid-based interventions for AD (Figure 1). It emphasizes their multi-target pharmacological actions across key pathological processes, including the restoration of skin barrier integrity, modulation of immune and chemokine dysregulation, itch signaling pathways, rebalancing of microbial homeostasis, modulation of programmed cell death pathways, and attenuation of oxidative stress and redox imbalance. Furthermore, the review highlights delivery innovations, safety considerations, and translational strategies, thereby providing a theoretical foundation for future research and clinical application of flavonoid-based therapies in AD. To ensure relevance and thematic focus, the following section outlines the literature search strategy underpinning this review.

## 2 Literature search strategy

To support the narrative synthesis, a focused literature search was conducted across PubMed, Web of Science, ScienceDirect, and SpringerLink, targeting English-language, peer-reviewed articles published between 2010 and 2025. The search strategy employed Boolean operators (e.g., AND, OR) to combine terms for natural flavonoid metabolites (e.g., quercetin, baicalin, epigallocatechin-3-gallate [EGCG]) with keywords related to AD and its underlying mechanisms, including skin barrier dysfunction, immune dysregulation, chemokine imbalance, pruritogenic signaling, microbial dysbiosis, programmed cell death, oxidative stress, and drug delivery.

We included *in vitro*, *in vivo*, and clinical studies that investigated the mechanisms of action, therapeutic relevance, or delivery approaches of flavonoids in AD models or patients. Articles were excluded if they centered on synthetic flavonoids, unrelated disease models, or lacked mechanistic or delivery-specific data. The review does not adhere to a systematic review framework but aims to offer a descriptive, theme-based synthesis of current evidence.

## 3 Classification and pharmacology of flavonoids

**FIGURE 2:**
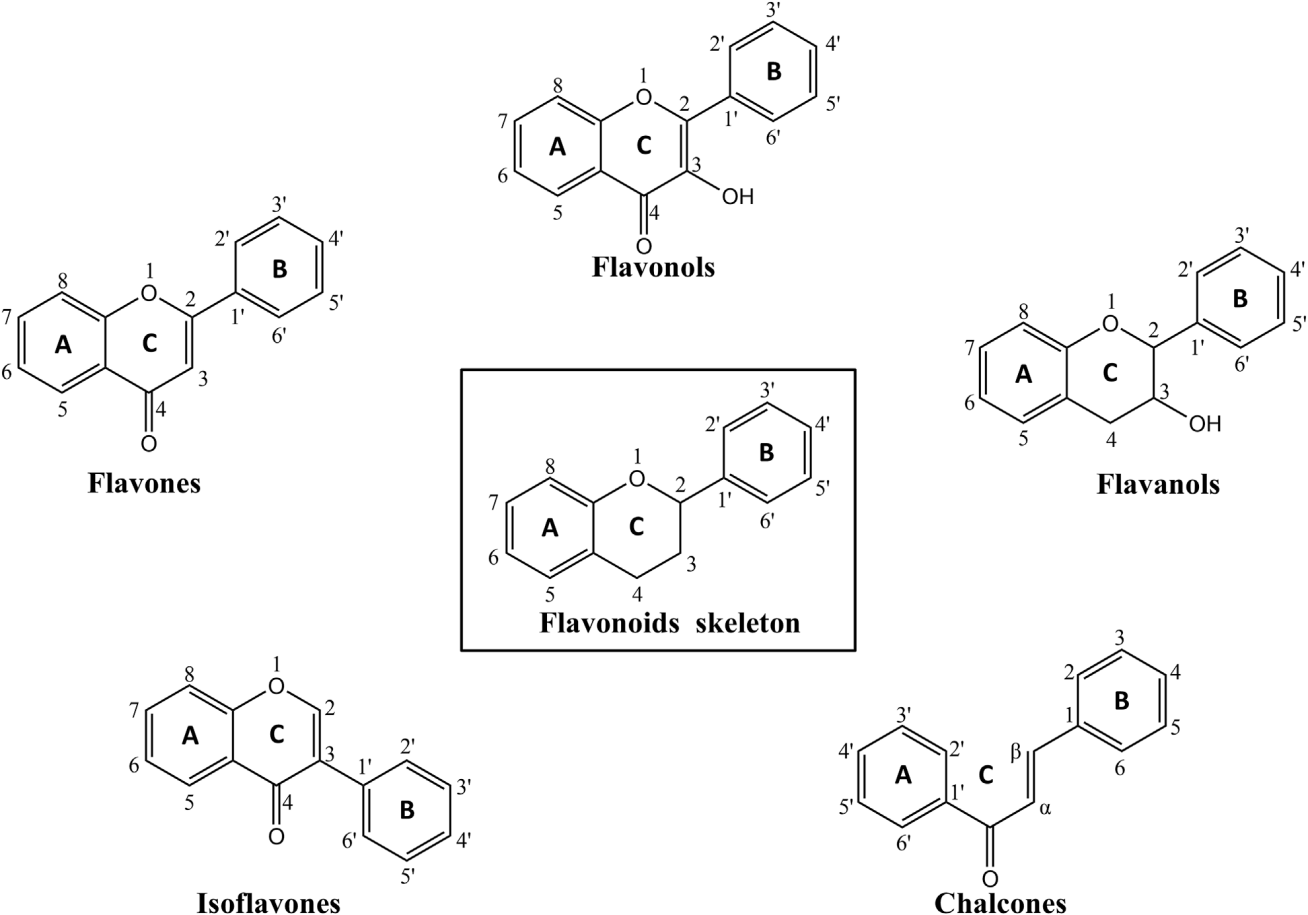
The chemical structures of flavonoids skeleton and corresponding subclasses involved in the paper. By KingDraw.

Flavonoids are defined by a characteristic C6–C3–C6 skeleton, comprising two aromatic rings connected by an oxygen-containing heterocyclic C-ring. Based on structural features, flavonoids are commonly categorized into several subgroups, including flavones, flavonols, flavanols, isoflavones, chalcones, etc. (Ku et al., 2020; Bo et al., 2024), as shown in Figure 2. Structural modifications such as the type and position of substituents and the degree of hydroxylation can directly influence the physicochemical properties, biological activities, and pharmacological profiles of different flavonoid subclasses (Zouaoui et al., 2021; Abou Baker, 2022).

Flavones are characterized by an unsaturated 2-phenylchromen-4-one backbone, with baicalin and luteolin being prominent representatives (Espíndola, 2023). These flavonoids have demonstrated the ability to reduce neuroinflammation and promote neural plasticity in diverse experimental models (Jin et al., 2019; Shi et al., 2021).

Flavonols feature a C3 hydroxyl group and a C2–C3 double bond, both of which enhance their hydroxyl radical scavenging capacity. These structural features allow flavonols to modulate oxidative stress and inflammation-related signaling pathways, resulting in both antioxidant and anti-inflammatory effects (Treml and Šmejkal, 2016). Representative flavonols such as quercetin and kaempferol have been shown to attenuate oxidative stress (Ji et al., 2015).

Flavanols are defined by multiple hydroxyl groups on the C-ring, with EGCG, a major green tea polyphenol, being a representative metabolite (Bernatoniene and Kopustinskiene, 2018; Payne et al., 2022). Functionally, EGCG selectively induces apoptosis and suppresses tumor cell proliferation (Kumazoe et al., 2022).

Isoflavones, a class of plant-derived phytoestrogens, are predominantly found in *Glycine max* (L.) Merr. (soybean), *Trifolium pratense* L. (red clover), and other leguminous plants (Messina, 2014). These flavonoid metabolites exert estrogen-like effects and can ameliorate postmenopausal osteoporosis by modulating estrogen receptor (ER)-mediated signaling pathways (Messina, 2016; Lambert and Jeppesen, 2018; Xiao et al., 2018).

Chalcones are open-chain flavonoids characterized by a 1,3-diphenylprop-2-en-1-one backbone and act as biosynthetic precursors to flavonoids and isoflavonoids. Their α, β-unsaturated carbonyl system contributes to their high reactivity and broad pharmacological potential (Tekale et al., 2020). Chalcones exhibit a wide range of biological activities, including anti-inflammatory, antioxidant, antimicrobial, and anticancer effects, largely through the modulation of signaling pathways such as nuclear factor kappa B (NF-κB), phosphoinositide 3-kinase/protein kinase B (PI3K/Akt), and cyclooxygenase (COX) enzymes (Ugwu et al., 2016).

## 4 Pharmacological effects of flavonoids in AD

**FIGURE 3:**
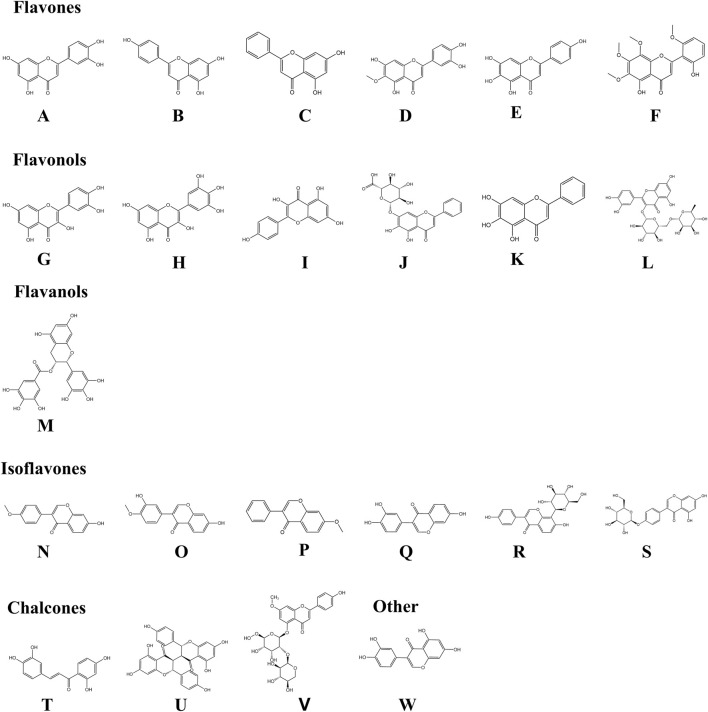
Chemical structures of flavonoids involved in the paper. By KingDraw. Among them, **(A)** Luteolin (Molecular formula: C_15_H_10_O_6_); **(B)** Apigenin (Molecular formula: C_15_H_10_O_5_); **(C)** Chrysin (Molecular formula: C_15_H_10_O_4_); **(D)** Nepetin (Molecular formula: C_16_H_12_O_7_); **(E)** Scutellarein (Molecular formula: C_15_H_10_O_6_); **(F)** Skullcapflavone II (Molecular formula: C_1_9H_18_O_8_); **(G)** Quercetin (Molecular formula: C_15_H_10_O_7_); **(H)** Myricetin (Molecular formula: C_15_H_10_O_8_); **(I)** Kaempferol (Molecular formula: C_15_H_10_O_6_); **(J)** Baicalin (Molecular formula: C_21_H_18_O_11_); **(K)** Baicalein (Molecular formula: C_15_H_10_O_5_); **(L)** Rutin (Molecular formula: C_27_H_30_O_16_); **(M)** Epigallocatechin-3-gallate (Molecular formula: C_22_H_18_O_11_); **(N)** Formononetin (Molecular formula: C_16_H_12_O_4_); **(O)** Calycosin (Molecular formula: C_16_H_12_O_5_); **(P)** 7-Methoxyisoflavone (Molecular formula: C_16_H_12_O_3_); **(Q)** 7,3′,4′-Trihydroxyisoflavone (Molecular formula: C_15_H_10_O_5_); **(R)** Puerarin (Molecular formula: C_21_H_20_O_9_); **(S)** Sophoricoside (Molecular formula: C_21_H_20_O_10_); **(T)** Butein (Molecular formula: C_15_H_12_O_5_); **(U)** Chamaejasmine (Molecular formula: C_30_H_22_O_10_); **(V)** Stechamone (Molecular formula: C_27_H_30_O_14_), and **(W)** Orobol (Molecular formula: C_15_H_10_O_6_).

Growing evidence indicates that numerous natural flavonoids (Figure 3) exert therapeutic effects in AD through multi-targeted pharmacological mechanisms (Table 1).

**TABLE 1:** Pharmacological effects of flavonoids in AD.

Metabolites	Methods	Models	Routes of administration	Targets	Signal pathways	Mechanisms	References
Flavones							
Luteolin	*In vivo*	NC/Nga mice, Kun Ming mice	Topical	Skin thickness↓, mast cell number↓, granulated mast cells infiltration↓, degranulated mast cells infiltration↓, mast cell degranulation↓, lymphocyte infiltration↓, skin hydration↑, Filaggrin↑, IL-4↓, IL-6↓, IL-17↓, TNF-α↓, IgE↓, IFN-γ↑, TEWL↓	JAK/STAT	Modulate immune imbalance, improve skin barrier function	Choi et al. (2010), Tang et al. (2022)
Apigenin	*In vitro*/*In vivo*	RAW264.7, RBL-2H3, HaCaT, HMC-1, SKH-1 hairless mice (hr/hr); ICR mice	Topical	Lamellar body↑, ABCA12↑, Filaggrin↑, TEWL↓, HMG-CoA reductase↑, SPT1↑, FAS↑, CAMP↑, mBD3↑, stratum corneum hydration↓, Loricrin↑, AQP3↑, HA↑, HAS-1↑, HAS-2↑, HAS-3↑, HBD-1↑, HBD-2↑, HBD-3↑, LL-37↑, NO↓, IL-1β↓, IL-6↓, COX-2↓, iNOS↓, β-hexosaminidase↓, TNF-α↓, IL-4↓, IL-5↓, IL-13↓, IL-31↓, Tryptase↓, FcεRIα↓, FcεRIγ↓, p-Lyn↓, p-Syk↓, p-PLCγ1↓	MAPK	Modulate immune imbalance, improve skin barrier function, attenuate pruritus, modulate skin and gut microbiota	Hou et al. (2013), Park et al. (2020a), Che et al. (2019)
Chrysin	*In vitro*/*In vivo*	HaCaT, BALB/c mice	Topical/Oral	Skin thickness↓, mast cell infiltration↓, CCL5+ cells↓, CCL5↓, IgE↓, TSLP↓, EGR1↓	NF-κB, MAPK	Regulate chemokines, attenuate pruritus	Yeo et al. (2020), Yeo et al. (2021)
Nepetin	*In vitro/In vivo*	HaCaT, BALB/c mice	Oral	Epidermal hyperplasia↓, immune cell infiltration↓, apoptosis rate↓, IL-1β↓, IL-6↓, TNF-α↓, ROS↓, iNOS↓, COX-2↓, PGES2↓, NO↓	MyD88–MKK3/6–Akt	Modulate oxidative stress, regulate programmed cell death	Gong et al. (2024)
Scutellarein	*In vitro*/*In vivo*	HaCaT, C57BL/6J mice	Subcutaneous injection	Skin thickness↓, TRPV3 current↓, TRPV3 open probability↓, TRPV3-mediated Ca^2+^ influx↓, BrdU incorporation↓, PBMCs chemotaxis↓, IgE↓, IL-1β↓, TNF-α↓, IL-4↓, IL-6↓, CXCL15↓	TRPV3 channels	Regulate chemokines, attenuate pruritus	Wang et al. (2022c)
Skullcapflavone II	*In* *vitro*/*In vivo*	BMDCs, Mouse CD4^+^ T cells, HaCaT, Human primary keratinocytes, BALB/c mice	Topical	Skin thickness↓, CD4^+^ T cell proliferation↓, CD4^+^ T cell infiltration↓, Gr-1^+^ neutrophil infiltration↓, mast cell infiltration↓, eosinophil infiltration↓, CTSS↓, pro-CTSS↓, active-CTSS↓, CXCL1↓, CCL17/22↓, TSLP↓, IL-4↓, IFN-γ↓, IL-6↓, IL-12↓, IL-17A↓, IgE↓, S100A8↓, Mcpt8↓	STAT, NF-κB, MAPK	Regulate chemokines, attenuate pruritus	Lee et al. (2022b)
Flavonols							
Quercetin	*In vitro/In vivo*	HaCaT, RAW264.7, human mast cells (HMC-1), NC/Nga mice, C57BL/6 mice	Topical/Oral	Skin thickness↓, mast cell infiltration↓, eosinophil levels↓, IL-1β↓, IL-1α↓, IL-4↓, IL-5↓, IL-6↓, IL-8↓, IL-10↑, IL-13↓, IL-15↓, IL-33↓, TNF-α↓, IFN-γ↓, IgE↓, TSLP↓, CCL2/3/5/7/11/17/22/27↓, TLR2↓, TLR6↓, HMGB1↓, COX-2↓, iNOS↓, ICAM-1↓, VEGF↓, caspase-3↓, caspase-8↓, Bid↓, Nrf2↑, HO-1↑, PPARα↑, PPARγ↑, glutathione↑, MMP-1↓, MMP-2↓, MMP-9↓, SOD1↑, SOD2↑, catalase↑, GPx↑, Twist↑, Snail↑, E-cadherin↑, Occludin↑	HMGB1/RAGE/NF-κB, Nrf2/HO-1, MAPK, JAK/STAT, TLR2/TLR6	Improve skin barrier function, regulate chemokines, modulate oxidative stress	Karuppagounder et al. (2016), Hou et al. (2019), Beken et al. (2020)
Myricetin	*In vitro/In vivo*	HaCaT, Balb/c Mice, Kunming mice	Topical	Skin thickness↓, mast cell infiltration↓, CD4^+^ T cell infiltration↓, keratinocyte integrity↑, lamellar body secretion area↑, TEWL↓, Filaggrin↑, IL-1β↓, IL-4↓, IL-17↓, IFN-γ↓, TNF-α↓, IgE↓, histamine↓, TSLP↓, CCL17/22↓, T-bet↓, GATA-3↓, TGF-β↓, iNOS↓, COX-2↓	NF-κB, STAT1	Improve skin barrier function, modulate immune imbalance	Hou et al. (2022), Gao et al. (2023)
Kaempferol	*In vitro/In vivo*	Jurkat T cells, CD4^+^ T cells and splenocytes from BALB/c mice, BALB/c mice, C57BL/6 mice	Oral/Intraperitoneal injection, i.p	Skin thickness↓, CD3^+^ T cells↓, CD4^+^ T cells↓, CD68^+^ macrophages↓, mast cell infiltration↓, TEWL↓, Filaggrin↑, Loricrin↑, Involucrin↑, IL-2↓, IL-4↓, IL-6↓, IL-13↓, IL-17↓, IL-31↓, IFN-γ↓, TSLP↓, CD69↓, cleaved caspase-3/7/9↓, MRP-1 activity↓, Bcl-2↑, HO-1↓	NF-κB, MAPK	Improve skin barrier function, modulate immune imbalance, regulate programmed cell death	Lee and Jeong (2021), Nasanbat et al. (2023)
Baicalin	*In vivo*	BALB/c mice, Pseudo germ-free mice	Oral	Skin thickness↓, mast cell infiltration↓, TEWL↓, TSLP↓, MCP-1↓, IL-4↓, IFN-γ↑, IgE↓, Filaggrin↑, Loricrin↑, Involucrin↑, histamine↓, *Alistipes* spp.↓, *Parabacteroides* spp.↓, *Mycoplasma* spp.↓, *Lactobacillus* spp.↑, *Coprococcus 1* spp.↑, *Ruminiclostridium 6* spp.↑	JAK/STAT, NF-κB	Improve skin barrier function, modulate immune imbalance, modulate skin and gut microbiota	Wang et al. (2022a)
Baicalein	*In vitro*	HaCaT	—	K1/K10↑	MAPK, Akt	Improve skin barrier function	Huang et al. (2016a)
Rutin	*In vivo*	BALB/c J mice	Topical	Skin thickness↓, eosinophil infiltration↓, mast cell infiltration↓, IL-4↓, IL-5↓, IL-13↓, IL-31↓, IL-32↓, IFN-γ↓, IgE↓, histamine↓	—	Modulate immune imbalance, attenuate pruritus	Choi and Kim (2013)
Flavanols							
Epigallocatechin-3-gallate	*In vivo*	Kunming mice, Nc/Nga mice	Topical	Skin thickness↓, mast cell number↓, IFN-γ↓, IL-4↓, IL-5↓, IL-6↓, IL-13↓, IL-17A↓, TNF-α↓, IgE↓, histamine↓, LDH activity↓, MDA↓, SOD↑, GSH↑, T-AOC↑, RIP1↓, RIP3↓, MLKL↓	NF-κB, MAPK	Modulate oxidative stress, regulate programmed cell death, modulate skin and gut microbiota	Chiu et al. (2021), Han et al. (2022)
Isoflavones							
Formononetin	*In vitro/In vivo*	HaCaT, RBL-2H3, BMMCs, BALB/c mice	Oral/Intraperitoneal injection, i.p	FcεRIγ, p-Lyn, p-Syk, p-PLCγ, IL-13, TNF-α, Filaggrin, Loricrin, IgE, β-hexosaminidase, histamine, TSLP, E-cadherin, IgE	NF-κB	Improve skin barrier function, attenuate pruritus	Li et al. (2018), Zhou et al. (2023)
Calycosin	*In vitro/In vivo*	HaCaT, C57BL/6 mice	Oral	Occludin↑, ZO-1↑, TSLP↓, IL-33↓	TLR4/MyD88/NF-κB	Improve skin barrier function, attenuate pruritus	Tao et al. (2017)
7-Methoxyisoflavone	*In vivo*	BALB/c mice	Topical	Skin thickness↓, keratinocyte hyperproliferation↓, mast cell infiltration↓, neutrophil infiltration↓, IL-17^+^ Th17 cells↓, spleen index↓, IL-4↓, IL-17A↓, IgE↓, IFN-γ↓, TSLP↓, CXCL1/2/3↓, CCL17/22↓	MAPK-AP-1, NF-κB, IL-17/STAT3	Modulate immune imbalance, regulate chemokines, attenuate pruritus	Dong et al. (2022)
7,3′,4′-Trihydroxyisoflavone	*In* *vitro/In vivo*	HaCaT, NC/Nga mice	Topical	Skin thickness↓, eosinophil infiltration↓, mast cell infiltration↓, IgE↓, CCL17/22↓	MAPK	Modulate oxidative stress, regulate chemokines	Park et al. (2020b)
Puerarin	*In vitro/In vivo*	HaCaT, BALB/c mice	Oral	Skin thickness↓, mast cell infiltration↓, IL-1β↓, IL-4↓, IL-5↓, IL-6↓, IL-31↓, TNF-α↓, IgE↓, PAR2↓, TSLP↓, CCL2/5/17↓, CXCL8/9/10/11↓	MAPK, NF-κB, Akt, STAT1, PAR2–NF-κB–TSLP axis	Modulate immune imbalance, regulate chemokines, attenuate pruritus	Lee et al. (2018)
Sophoricoside	*In vitro/In vivo*	C57BL/6 mice naïve CD4^+^ T, BALB/c mice	Topical	Skin thickness↓, mast cell infiltration↓, IgE↓, IFN-γ↓, TNF-α↓, IL-2↓, IL-4↓, IL-5↓, IL-6↓, IL-12↓, IL-17A↓, T-bet↓, GATA-3↓, RORγt↓	—	Modulate immune imbalance, attenuate pruritus	Kim and Lee (2021)
Chalcones							
Butein	*In vitro*	HaCaT, human acute monocytic leukemia cell line THP-1 (THP-1 cells)	Topical	ICAM-1↓, monocyte adhesion↓, IL-6↓, IP-10↓, MCP-1↓, ROS↓	MAPK, NF-κB	Modulate oxidative stress	Seo et al. (2015)
Chamaejasmine	*In vitro/In vivo*	RBL-2H3, SKH-1 hairless mice	Topical	Skin thickness↓, skin hydration↑, TEWL↓, mast cells↓, IgE↓, IL-4↓, β-hexosaminidase↓	—	Improve skin barrier function, modulate immune imbalance, attenuate pruritus	Kim et al. (2019)
Stechamone	*In vitro*/*In vivo*	RBL-2H3, SKH-1 hairless mice	Topical	Skin thickness↓, mast cell↓, lymphocyte infiltration↓, TEWL↓, β-hexosaminidase↓, IgE↓, IL-4↓	—	Improve skin barrier function, modulate immune imbalance, attenuate pruritus	Jo et al. (2018)
Others							
Orobol	*In vitro*/*In vivo*	HaCaT, NC/Nga mice	Topical	Skin thickness↓, skin hydration↑, TEWL↓, eosinophil infiltration↓, mast cell infiltration↓, CCL17/22↓, IgE↓, IL-4↓, IL-13↓	MAPK, NF-κB	Improve skin barrier function, regulate chemokines, modulate immune imbalance, attenuate pruritus	Lee et al. (2022a)

Note: BMDCs, bone marrow-derived dendritic cells; BMMCs, bone marrow-derived mast cells; CD4^+^ T cells, cluster of differentiation 4 positive T lymphocytes; HaCaT, human immortalized keratinocyte cell line; HMC-1, human mast cell line-1; Jurkat T cells, human T lymphocyte leukemia cell line; RBL-2H3, rat basophilic leukemia cells; RAW264.7, murine macrophage-like cell line; SKH-1, hairless mice (hr/hr), hairless mouse strain used for dermatological research; ICR, mice, Institute of Cancer Research mice; THP-1, human acute monocytic leukemia cell line; ABCA12, ATP-binding cassette subfamily A member 12; AQP3, aquaporin 3; Bcl-2, B-cell lymphoma 2; Bid, BH3-interacting domain death agonist; BrdU, 5-bromo-2′-deoxyuridine; CAMP, cathelicidin antimicrobial peptide; CAT, catalase; CCL, C-C motif chemokine ligand; CD69, cluster of differentiation 69; COX-2, cyclooxygenase-2; CTSS, Cathepsin S; CXCL-, C-X-C motif chemokine ligand; EGR1, early growth response 1; FAS, fatty acid synthase; FcεRIα, high-affinity IgE receptor subunit alpha; FcεRIγ, high-affinity IgE receptor subunit gamma; GATA-3, GATA-binding protein 3; GPx, glutathione peroxidase; GSH, glutathione; HAS, hyaluronic acid synthase; HBD-, human beta-defensin; HMGB1, high mobility group box 1; HO-1, heme oxygenase-1; ICAM-1, intercellular adhesion molecule 1; IFN-γ, interferon-gamma; IgE, immunoglobulin E; IL-, interleukin; iNOS, inducible nitric oxide synthase; K1/K10, keratin 1 and keratin 10; LDH, lactate dehydrogenase; MAPK, mitogen-activated protein kinase; MCP-1, monocyte chemoattractant protein 1; MDA, malondialdehyde; Mcpt8, mast cell protease 8; MLKL, mixed lineage kinase domain-like protein; mBD3, mouse beta-defensin 3; MMP-, matrix metalloproteinases, MRP-1, multidrug resistance-associated protein 1; MyD88, myeloid differentiation primary response 88; NO, nitric oxide; Nrf2, nuclear factor erythroid 2-related factor 2; Occludin, tight junction protein occludin; PAR2, protease-activated receptor 2; PGES2, prostaglandin E synthase 2; PGE_2_, prostaglandin E_2_; p-Lyn, phosphorylated Lyn; p-Syk, phosphorylated spleen tyrosine kinase; p-PLCγ1, phosphorylated phospholipase C gamma 1; PPARα, peroxisome proliferator-activated receptor alpha; PPARγ, peroxisome proliferator-activated receptor gamma; RAGE, receptor for advanced glycation end-products; RIP1, receptor-interacting serine/threonine-protein kinase 1; RIP3, receptor-interacting serine/threonine-protein kinase 3; RORγt, RAR-related orphan receptor gamma t; ROS, reactive oxygen species; S100A8, S100 calcium-binding protein A8; Snail, zinc finger protein SNAI1; SOD, superoxide dismutase; SPT1, serine-palmitoyl transferase 1; STAT, signal transducer and activator of transcription; TEWL, transepidermal water loss; T-bet, T-box transcription factor (TBX21); TGF-β, transforming growth factor beta; TLR-, Toll-like receptor; TNF-α, tumor necrosis factor-alpha; TRPV3, transient receptor potential vanilloid 3; TSLP, thymic stromal lymphopoietin; Twist, twist-related protein 1; VEGF, vascular endothelial growth factor; ZO-, zonula occludens; Akt, protein kinase B; AP-1, activator protein 1; JAK, janus kinase; MAPK, mitogen-activated protein kinase; MKK3/6, mitogen-activated protein kinase kinase 3/6; MyD88, myeloid differentiation primary response 88; NF-κB, nuclear factor kappa B; TRPV3, transient receptor potential vanilloid 3 channel.

### 4.1 Restoration of skin barrier integrity

**FIGURE 4:**
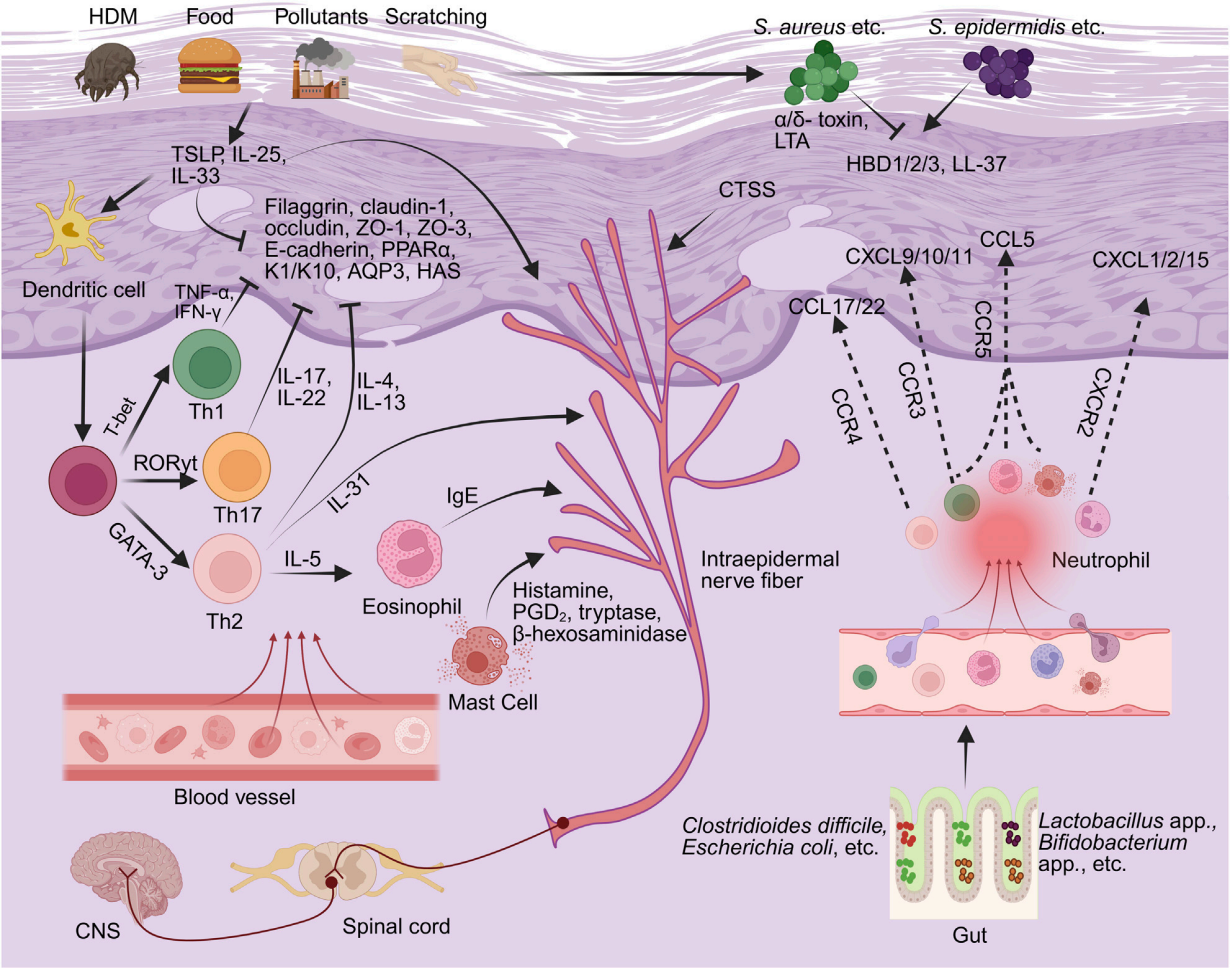
The pathological mechanisms underlying AD (the part that flavonoids can intervene in). By BioRender. Note: HDM, house dust mite; TSLP, thymic stromal lymphopoietin; IL-, interleukin; IFN-γ, interferon-gamma; TNF-α, tumor necrosis factor-alpha; ZO-, Zonula Occludens-; K1/K10, keratin 1 and keratin 10; PPARα, peroxisome proliferator-activated receptor alpha; AQP3, aquaporin 3; HAS, hyaluronic acid synthase; Th-, T helper; T-bet, T-box transcription factor (TBX21); GATA3, GATA-binding protein 3; RORγt, retinoic acid-related orphan receptor gamma t; CCR-, C-C chemokine receptor; CCL, C-C motif chemokine ligand; CXCL-, C-X-C motif chemokine ligand; HBD, human beta-defensin; *Staphylococcus aureus*, *Staphylococcus aureus*; *Staphylococcus epidermidis*, *Staphylococcus epidermidis*; LTA, lipoteichoic acid; IgE, immunoglobulin E; PGD_2_, prostaglandin D_2_; CGRP, calcitonin gene-related peptide; CTSS, Cathepsin S.

Disruption of the skin barrier, comprising the stratum corneum, lamellar lipids, tight junctions, and cutaneous microbiota, is a central pathophysiological hallmark of AD, as part of the broader mechanisms illustrated in Figure 4. This dysfunction is characterized by downregulated structural protein expression, disorganized intercellular junctions, lipid imbalance, and microbial dysbiosis (Melnik, 2015; Fujii, 2020). These alterations result in increased transepidermal water loss (TEWL) and a diminished protective barrier, which together permit the transcutaneous entry of allergens and pro-inflammatory mediators. Consequently, keratinocyte hyperactivation and immune dysregulation are initiated, establishing a self-perpetuating cycle of barrier disruption and inflammatory amplification that ultimately drives disease progression (Katsarou et al., 2023; Çetinarslan et al., 2023).

At the molecular level, AD skin lesions exhibit significant downregulation of structural proteins such as filaggrin and claudin-1 (Melnik, 2015; Callou et al., 2022), as well as tight junction-associated proteins, among which Zonula Occludens- (ZO-) 1 and occludin shown to be significantly reduced in AD skin lesions (Yuki et al., 2016), and ZO-3 expression diminished in keratinocyte-based AD models (Hu et al., 2023; Trujillo-Paez et al., 2024). These alterations collectively result in impaired keratinocyte cohesion and elevated TEWL. Notably, these alterations occur prior to the onset of inflammation and induce the upregulation of epithelial-derived cytokines such as thymic stromal lymphopoietin (TSLP) and interleukin-33 (IL-33), which subsequently activate the Th2 immune axis in a sustained manner (Dai et al., 2022). Several flavonoid metabolites have demonstrated potential to modulate barrier function through multiple mechanisms. Chamaejasmine, stechamone, and orobol have been shown to reduce TEWL, increase skin hydration, mitigate erythema, pruritus, and xerosis, attenuate epidermal hyperplasia and mast cell infiltration, and attenuate stratum corneum damage (Jo et al., 2018; Kim et al., 2019; Lee C. H. et al., 2022). Baicalin and formononetin significantly upregulate the expression of filaggrin and loricrin, reduce TEWL, suppress mast cell infiltration, and ameliorate both epidermal disruption and modified Eczema Area and Severity Index (EASI) scores in AD mouse models (Wang L. et al., 2022; Zhou et al., 2023). Experimental studies indicate that calycosin may modulate the Toll-like receptor (TLR) 4/NF-κB signaling pathway, suppress the expression of TSLP and IL-33, and increase tight junction proteins such as ZO-1 and occludin in both human immortalized keratinocyte cell line (HaCaT) and mouse AD models (Tao et al., 2017).

Adherens junctions (AJs) play a critical role in maintaining epidermal integrity, with E-cadherin serving as a central adhesion molecule. In AD, downregulation of E-cadherin disrupts intercellular cohesion, resulting in elevated TEWL and increased permeability to environmental allergens (Dong et al., 2024). In a fluorescein isothiocyanate (FITC)-induced AD mouse model, formononetin was observed to improve E-cadherin localization, suppress the expression of TSLP and IL-33, reinforce the epidermal barrier integrity, and attenuate immune activation (Li et al., 2018). In HaCaT model, quercetin treatment was associated with increased expression of E-cadherin and occludin, upregulation of antioxidant enzymes including superoxide dismutase 1 (SOD1) and superoxide dismutase 2 (SOD2) and catalase, and reduced levels of pro-inflammatory cytokines and matrix metalloproteinases (MMPs), which may support improved keratinocyte migration and *in vitro* wound closure (Beken et al., 2020).

At the cytoskeletal level, keratins keratin 1 and keratin 10 (K1/K10) are critical structural proteins that contribute to the maintenance of epidermal mechanical integrity and barrier stability; however, their expression is markedly suppressed in AD skin lesions (Dai et al., 2021). In HaCaT model, baicalein was associated with promoting the structural maturation of keratinocytes by upregulating the expression of K1/K10 (Huang K.-F. et al., 2016).

Lamellar bodies, specialized organelles in the granular layer, secrete polar lipids and enzymes to form the stratum corneum’s intercellular lipid matrix (Elias and Wakefield, 2020; Shin et al., 2020); their impaired formation contributes to barrier dysfunction in AD (Cork et al., 2009). In addition, aquaporin 3 (AQP3) and hyaluronic acid synthase (HAS) are essential regulators of skin hydration and hyaluronic acid synthesis (Camilion et al., 2022; Tricarico et al., 2022). Myricetin has been reported to increase lamellar body secretion and support the structural integrity of the epidermal lipid matrix in AD mouse model (Gao et al., 2023), while apigenin has been reported to increase the expression of AQP3, HAS, and filaggrin, upregulates skin hydration and lipid synthesis, increase lamellar body density, and increase the expression of lipid-synthesizing enzymes, indicating potential roles in epidermal hydration and barrier maintenance in cell models (Hou et al., 2013; Park C.-H. et al., 2020).

Peroxisome proliferator-activated receptor alpha (PPARα), a key nuclear transcription factor that regulates keratinocyte differentiation and epidermal lipid metabolism, is markedly downregulated in AD (Kuai et al., 2024), leading to impaired barrier integrity and defective lipid synthesis, which further supported by PPARα agonist-induced barrier restoration in *ex vivo* skin model (Majewski et al., 2021). In a house dust mite-induced NC/Nga mouse model (a spontaneous AD model), quercetin has been reported to activate the PPARα signaling pathway, which may contribute to the improvements in lipid biosynthesis, inflammatory status, and barrier integrity (Karuppagounder et al., 2016).

### 4.2 Modulation of immune dysregulation

CD4^+^ T lymphocytes (CD4^+^ T cells) are central to adaptive immunity and differentiate into functionally subsets such as T helper (Th)1, Th2, Th17, and regulatory T cells (Tregs), which mediate distinct immune responses. These lineages maintain immune homeostasis through cytokine- and transcription factor-driven cross-regulation. Among these, Th1, Th2, and Th17 subsets are particularly implicated in the initiation and chronic progression of AD (Su et al., 2017), as part of the broader mechanisms illustrated in Figure 4.

In AD, an imbalance between Th1 and Th2 cells is recognized as one of the most prominent immunological hallmarks. Th1 cells differentiate in response to IL-12 and interferon-gamma (IFN-γ), a process regulated by the transcription factor T-bet (Jankovic and Feng, 2015). These cells predominantly secrete IFN-γ and tumor necrosis factor-alpha (TNF-α). In contrast, Th2 cells are induced by IL-4, which promotes the expression of GATA-binding protein 3 (GATA3) and leads to the production of IL-4, IL-5, and IL-13, all of which play key roles in driving allergic inflammation (Ruterbusch et al., 2020). During the acute phase of AD, Th2 cytokines are markedly upregulated, facilitating immunoglobulin E (IgE) production, eosinophil infiltration, and suppression of Th1 responses, thereby amplifying Th1/Th2 imbalance (Spergel et al., 1999). Among these, IL-4 and IL-13 have been identified as central pathogenic mediators that induce inflammation, impair skin barrier integrity, and provoke pruritus (Sehra et al., 2010; Bieber, 2020). In the chronic phase, Th1-related activity becomes predominant, characterized by increased production of IFN-γ and TNF-α (Moss et al., 2004), which further contribute to tissue damage and sustained immune dysregulation (Salama and Fowell, 2020). Several flavonoids have been reported to restore Th1/Th2 balance by targeting key signaling pathways. Apigenin has been reported to modulate allergic responses in both *in vitro* and *in vivo* models. In IgE-sensitized rat basophilic leukemia cells (RBL-2H3) cell model, apigenin treatment was associated with the suppression of FcεRI-mediated activation and downstream MAPK signaling, accompanied by reduced levels of Th2 cytokines (IL-4, IL-5, IL-6, IL-13) and TNF-α (Park C.-H. et al., 2020). Complementary findings in a compound 48/80-induced AD mouse model demonstrated attenuation of mast cell activation and IL-31-associated signaling pathways (Che et al., 2019). Similarly, in HaCaT model, myricetin treatment was associated with the downregulation of T-bet and GATA3 expression, accompanied by decreased levels of IL-4 and IFN-γ, suggesting a potential role in modulating Th1/Th2-associated immune signaling (Hou et al., 2022).

In addition to Th1 and Th2 subsets, Th17 cells and their associated cytokines are also involved in the immunopathogenesis of AD. In acute AD lesions, Th17-derived cytokines such as IL-17 and IL-22 are markedly elevated (Simon et al., 2014), which aggravates skin inflammation and disrupts epidermal barrier integrity (Asarch et al., 2008; Korn et al., 2009). The differentiation of Th17 cells is regulated by the lineage-specific transcription factor retinoic acid-related orphan receptor gamma t (RORγt) (Zhao et al., 2021; Hall et al., 2022). Luteolin has been observed to inhibit the Janus kinase 2 (JAK2)/STAT3 signaling pathway in AD mouse model, along with reduced IL-17 expression and diminished Th17-mediated inflammatory responses (Tang et al., 2022). Experimental evidence from AD mouse models suggests that 7-methoxyisoflavone exhibits dual immunomodulatory effects in both Th2- and Th17-predominant settings. In the Th2-driven FITC-induced model, it is associated with the downregulation of IL-4 and IFN-γ, while in the Th17-driven oxazolone (OXZ) model, it suppresses phosphorylated STAT3 (p-STAT3) and IL-17A expression, ultimately contributing to the coordinated regulation of Th1, Th2, and Th17 immune axes (Dong et al., 2022). Experimental studies integrating both *in vitro* and *in vivo* models have investigated the immunomodulatory properties of sophoricoside. *In vitro*, sophoricoside was shown to inhibit CD4^+^ T cell differentiation into Th1 (T-bet), Th2 (GATA3), and Th17 (RORγt) subsets, accompanied by reduced expression of IFN-γ, IL-4, and IL-17A. In a corresponding AD mouse model, sophoricoside treatment was associated with attenuated inflammatory cell infiltration and cytokine production in lesional skin, thereby mitigating AD-like pathological manifestations (Kim and Lee, 2021).

### 4.3 Modulation of chemokine dysregulation

Chemokines are small, secreted proteins produced by both structural and immune cells that regulate immune responses at sites of inflammation by directing immune cell migration through interactions with G protein-coupled receptors, including CC chemokine receptors (CCR) and CXC chemokine receptors (CXCR) (Liu J. et al., 2021). In AD, chemokines serve as critical mediators bridging innate and adaptive immunity (Sun et al., 2021), as part of the broader mechanisms illustrated in Figure 4. They coordinate the temporal recruitment of immune cell subsets and facilitate the progression from acute to chronic inflammation (Nedoszytko et al., 2014). In addition, chemokines play essential roles in driving Th2-skewed immune responses, disrupting skin barrier function, and amplifying downstream inflammatory signaling cascades (Kitahata et al., 2022).

During the acute phase of AD, Th2-associated chemokines play a dominant role in promoting inflammation. C-C motif chemokine ligand 17 and 22 (CCL17 and CCL22), predominantly secreted by keratinocytes and dendritic cells, bind to C-C chemokine receptor 4 (CCR4) and selectively recruit Th2 cells to lesional skin. These chemokines are widely recognized as key biomarkers of disease activity (Carola et al., 2021; He et al., 2021). Quercetin has been reported to attenuate Th2-mediated inflammation in MC903-induced AD mouse model along with reduced expression of CCL17 and CCL22, which may contribute to reduced Th2 cell infiltration. In HaCaT model, quercetin treatment was associated with decreased secretion of CCL17 and CCL22 secretion, potentially through modulation of the TLR2/6 and MAPK signaling pathways (Hou et al., 2019). Similarly, other flavonoids such as 7,3′,4′-trihydroxyisoflavone, skullcapflavone II, and orobol have been reported to downregulate CCL17 and CCL22 expression both in mouse and HaCaT AD models. These effects appear to be primarily associated with the modulation of signaling pathways such as MAPK, NF-κB, and STAT. By interfering with chemokine-mediated immune cell recruitment, these flavonoid metabolites have been associated with attenuated cutaneous inflammation, including erythema, pruritus, and barrier disruption (Park S. H. et al., 2020; Lee Y. et al., 2022; Lee et al., 2022 C. H.).

In addition to Th2-associated chemokines, CCL5 is also significantly upregulated in AD, although it is not Th2-specific. This chemokine acts on Th1 cells, eosinophils, and mast cells to promote immune cell infiltration and epidermal hyperplasia, which functions as a mediator linking acute and chronic phases, as supported by its upregulation across disease stages (Yu et al., 2020; Matsui et al., 2022). Recent studies have reported that chrysin mitigates inflammatory cell infiltration and histopathological alterations in AD mouse model, potentially via suppression of the NF-κB signaling pathway and downregulation of CCL5 expression (Yeo et al., 2020).

As inflammation progresses, neutrophil-associated CXC chemokines play an increasingly prominent role in the pathogenesis of AD. Among them, C-X-C motif chemokine ligand 1 and 2 (CXCL1 and CXCL2) are significantly upregulated in lesional skin and primarily mediate neutrophil recruitment through interaction with the CXCR2 receptor, thereby aggravating tissue damage and acute symptoms such as erythema and edema (Sakai et al., 2021). Transcriptomic analyses by Chen et al. revealed that CXCL1 and CXCL2 are highly enriched in IL-17-related signaling pathways, which participate in acute immune responses triggered by microbial coinfections (Chen et al., 2019). Experimental evidence indicates that skullcapflavone II downregulates CXCL1 expression in MC903-induced AD mouse model, which is associated with reduced infiltration of CD4^+^ T cells, eosinophils, and neutrophils (Lee Y. et al., 2022). In addition, 7-methoxyisoflavone has been reported to significantly suppress CXCL1, CXCL2, and CXCL3 expression in both FITC- and oxazolone-induced AD models, which was associated with reduced neutrophil infiltration and attenuated skin erythema and edema (Dong et al., 2022).

CXCL15, a murine neutrophil chemotactic factor, is markedly upregulated during the early inflammatory stage in 2,4-dinitrochlorobenzene (DNCB)-induced model of AD. It acts synergistically with CXCL1 and CXCL2 to promote neutrophil infiltration and enhance inflammatory cell accumulation in the skin (Zheng et al., 2023). In carvacrol- and 2,4-dinitrofluorobenzene (DNFB)-induced mouse models, scutellarein treatment was associated with reduced secretion of CXCL15 and related pro-inflammatory cytokines, thereby alleviating AD-like symptoms (Wang Y. et al., 2022).

As AD progresses into the chronic phase, Th1-mediated immune responses become increasingly dominant, characterized by the sustained overexpression of IFN-γ-induced chemokines such as CXCL9, CXCL10, and CXCL11. These chemokines play a critical role in maintaining chronic inflammation and driving tissue damage (Drozhdina and Suslova, 2021). Studies by Renert-Yuval et al. (2021) and Kibalina et al. (2022) have shown that CXCL9, CXCL10, and CXCL11 are significantly upregulated and co-expressed with IFN-γ in the lesional skin of adult AD patients, further substantiating their role in the Th1-dominant inflammatory axis. Puerarin has been reported to suppress the expression of Th1-associated chemokine (CXCL9, CXCL10, CXCL11) in TNF-α/IFN-γ-stimulated HaCaT model, and to attenuate inflammation and skin tissue damage in a DNCB-induced AD mouse model (Lee et al., 2018).

### 4.4 Modulation of itch signaling pathways

**FIGURE 5:**
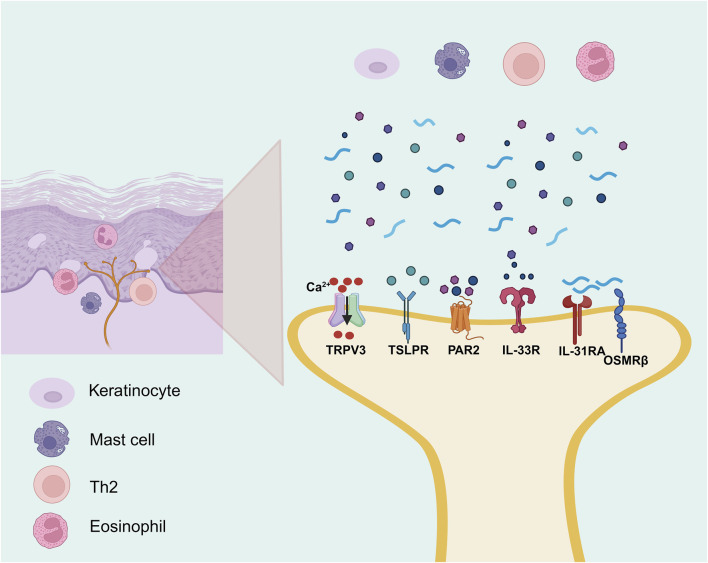
The itch signaling underlying AD (the part that flavonoids can intervene in). By BioRender. Note: Th2, T helper 2 cell; TSLPR, thymic stromal lymphopoietin receptor; IL-13RA, interleukin-13 receptor alpha; OSMRβ, oncostatin M receptor beta; IL-33R, interleukin-33 receptor; PAR2, protease-activated receptor-2; TRPV3, transient receptor potential vanilloid 3.

Persistent or recurrent pruritus is a hallmark symptom of AD, and its pathogenesis is driven by complex interactions among upstream inflammatory mediators, neuronal sensitization mechanisms, and immune-driven processes (Tominaga and Takamori, 2022). Recent evidence suggests that AD-associated itch is mediated by a broad spectrum of signaling molecules, including pro-inflammatory cytokines such as IL-31 (Duca et al., 2022), IL-33 (Nakajima et al., 2021) and TSLP (Meng J. et al., 2021); sensory ion channels such as members of the transient receptor potential (TRP) family (Meng J. et al., 2021); immune effector events such as IgE-mediated mast cell degranulation (Mollanazar et al., 2016); and proteolytic mediators including Cathepsin S (CTSS) (Ruppenstein et al., 2021). These factors may act synergistically amplify peripheral itch perception and contribute to the persistence and exacerbation of chronic pruritus through multiple signaling cascades, as partially illustrated in Figure 4 and in greater detail in Figure 5.

TSLP and IL-33 serve as upstream alarmins in AD, initiating both immune and sensory signaling. TSLP is abundantly expressed in keratinocytes of AD lesions and promotes Th2-type inflammation while directly activating transient receptor potential ankyrin 1 (TRPA1) channels on sensory neurons, enhancing pruritus (Wilson et al., 2013; Meng J. et al., 2021). It also upregulates IL-33 and synergizes to suppress barrier proteins such as filaggrin and claudin-1, exacerbating epidermal dysfunction (Dai et al., 2022). Upon cellular damage, IL-33 activates type 2 innate lymphoid cells (ILC2s) and basophils to produce IL-5 and IL-13, and simultaneously enhances IL-31 signaling, forming a self-reinforcing loop of inflammation and itch (Imai, 2023; Yamamura et al., 2024). As a Th2 effector, IL-31 correlates with itch severity and signals via IL-31 receptor alpha (IL-31RA)/Oncostatin M receptor beta (OSMRβ) receptors on sensory nerves to release calcitonin gene-related peptide and drive neurogenic inflammation (Fassett et al., 2023). Several flavonoids have been reported exhibit pharmacological effects in alleviating itch-associated inflammatory responses in AD models. In DNCB-induced AD mouse model, puerarin treatment was associated with reduced expression of pruritogenic cytokines including IL-31 and TSLP, indicating its potential anti-inflammatory activity (Lee et al., 2018). In addition, in a *Dermatophagoides farinae* extract and DNCB-induced AD mouse model, rutin was reported to downregulate Th2-type cytokines including IL-31, IL-4, and IL-13, along with decreased serum histamine levels and suppressing mast cell infiltration in lesional skin (Choi and Kim, 2013).

In addition to classical Th2 cytokines, non-canonical sensory pathways are essential contributors to chronic pruritus in AD. CTSS, a protease strongly associated with itch, is markedly upregulated in AD lesions and sustains neuroimmune activation by cleaving PAR2 or directly stimulating sensory nerve endings (Ruppenstein et al., 2021). In parallel, the transient receptor potential vanilloid 3 (TRPV3) calcium channel, overexpressed in keratinocytes and sensory neurons, increases neuronal excitability and contributes to persistent itch (Meng J. et al., 2021). In HaCaT model, skullcapflavone II was reported to suppress the expression of CTSS, a pruritogenic protease, through inhibition of the STAT1/NF-κB/p38 MAPK signaling pathway (Lee Y. et al., 2022). Likewise, in AD mouse model, scutellarein was reported to inhibit TRPV3 channel activity, accompanied by reduced, accompanied by reduced neural hyperexcitability, lower serum IgE levels, and decreased expression of proinflammatory cytokines including IL-1β, TNF-α, IL-4, IL-6, and CXCL15 (Wang Y. et al., 2022).

During the acute phase of AD, the interplay between immune cells and the peripheral nervous system plays a pivotal role in initiating and amplifying pruritus (Steinhoff et al., 2022). Among these mechanisms, IgE-mediated mast cell degranulation serves as a key driver of immediate itch responses. Upon activation, mast cells rapidly release pruritogenic mediators such as histamine, prostaglandin D_2_ (PGD_2_), tryptase, and β-hexosaminidase (Poto et al., 2022), which collectively stimulate C-type sensory nerve fibers and trigger neuroimmune amplification (Siiskonen and Harvima, 2019). Tryptase can further potentiate itch signal transmission by activating sensory neurons through PAR2. Several natural metabolites have shown inhibitory effects on mast cell-mediated pruritic signaling. For instance, *Daphnopsis costaricensis* Barringer and Grayum extract identified with 11 flavonoid metabolites and stechamone were observed to suppresse β-hexosaminidase release, reduce mast cell degranulation and IL-4 production, and were associated with attenuated scratching behavior in mouse AD models (Jo et al., 2018; Bae et al., 2022). Chamaejasmine was also observed to attenuate degranulation in IgE-sensitized RBL-2H3 and was associated with reduced serum levels of histamine, IgE, and IL-4 in AD mouse model (Jo et al., 2018; Kim et al., 2019). Moreover, luteolin has been shown to reduce serum IgE levels and attenuate scratching behavior in AD mouse model (Choi et al., 2010), with additional evidence showing its pharmacological effects in various pruritus models suggests that luteolin may inhibit mast cell degranulation and suppression of IL-4/IgE and PGD_2_–IL-33 signaling pathways (Gendrisch et al., 2021).

### 4.5 Regulation of skin and gut microbial homeostasis

In AD, the disruption of skin and gut microbial homeostasis is increasingly recognized as a critical factor contributing to the initiation and persistence of chronic inflammation and epithelial barrier dysfunction (Pessôa et al., 2023; Chen et al., 2024), as part of the broader mechanisms illustrated in Figure 4. Under normal conditions, commensal skin microbiota help preserve homeostasis by regulating cutaneous pH, promoting barrier protein expression, and shaping local immune responses (Naik et al., 2012). Among them, *Staphylococcus epidermidis* and other resident species strengthen skin integrity and immune tolerance by producing antimicrobial peptides, maintaining an acidic microenvironment, and preventing pathogenic colonization (Olesen et al., 2021).

In patients with AD, cutaneous microbial diversity is markedly reduced, often accompanied by predominant colonization by *S. aureus* (*Staphylococcus aureus*), which contributes significantly to recurrent inflammation and skin barrier dysfunction (Demessant-Flavigny et al., 2023). Notably, the hands, as characteristically dry sites, harbor distinct microbial communities that help maintain acidic pH and microbial balance (Huang et al., 2025); however, repeated scratching disrupts this equilibrium, promotes pH neutralization, and facilitates *S. aureus* adhesion and inflammatory responses (Bay et al., 2025).

*Staphylococcus aureus* in AD skin has also been shown to accumulate in keratinocyte lysosomes and trigger IL-1α secretion via TLR9 activation, thereby promoting inflammation (Moriwaki et al., 2019). It also secretes multiple virulence factors such as superantigens, α- and δ-toxins, and lipoteichoic acid (LTA), which activate T cells to produce IL-4, IL-5, TSLP, and IL-31 (Chung et al., 2022); collectively promote IgE class switching and Th2 polarization (Gough et al., 2022); and contribute to heightened pruritus and inflammation in AD (Aziz et al., 2024). In parallel, *S. aureus* downregulates the expression of key barrier-associated proteins such as filaggrin and loricrin (Cau et al., 2021), compromising keratinocyte cohesion and exacerbating skin barrier dysfunction (Demessant-Flavigny et al., 2023). A mechanism-oriented systematic review identified 85 flavonoid metabolites with anti-MRSA activity, approximately 70% of which demonstrated potent *in vitro* pharmacological effects (MIC ≤16 μg/mL) via membrane disruption, biofilm inhibition, and efflux pump suppression, suggesting their potential suitability for topical application in managing *S. aureus*-associated skin microbiota dysbiosis (Xu et al., 2024). Among them, a study reported that pure EGCG, at a concentration of 80 μg/mL, achieved a 99.999% (log_5_) reduction in clinical MRSA isolates within 4 h, indicating potent antibacterial effects (Aljuffali et al., 2022; Feilcke et al., 2023). Given EGCG’s multi-target pharmacological effects in AD models (Śladowska et al., 2025), incorporation of an *S. aureus*-induced model may facilitate investigation into its potential microbiota-modulating mechanisms. This model is characterized by pronounced Th1/Th17 inflammation and microbial dysbiosis, thereby providing a relevant experimental platform to examine how flavonoids contribute to restoring gut–skin axis homeostasis in AD (Zhang et al., 2024).

In addition to its local cutaneous effects, gut microbiota dysbiosis plays a significant role in systemic immune regulation via the gut–skin axis (Melli et al., 2020). Patients with AD frequently exhibit reduced gut microbial diversity (Melli et al., 2020), characterized by decreased abundance of beneficial genera such as *Lactobacillus* app. and *Bifidobacterium* app., along with increased abundance of pro-inflammatory taxa including *Clostridioides difficile* (Ahn, 2023). This dysbiosis leads to impaired production of short-chain fatty acids, disruption of the intestinal epithelial barrier (Pessôa et al., 2023), reduced differentiation of Tregs, and exaggerated Th2-type immune responses, all of which contribute to heightened skin sensitivity and inflammation (Chen et al., 2024). Emerging studies have demonstrated that glycosylated flavonoids exert microbiota-modulatory effects by selectively enriching beneficial bacterial taxa and suppressing pathogenic bacteria, thereby contributing to the reestablishment of gut microbial homeostasis (Pan et al., 2023; Xiong et al., 2023). Recent study suggests that the flavonoid baicalin may modulate gut microbiota composition and is associated with the alleviation of AD-like phenotypes. In DNCB-induced AD mouse model, baicalin was found to modulate gut microbial balance by increasing the abundance of *Lactobacillus* app. and *Coprococcus 1* app., while reducing *Parabacteroides* app. and *Alistipes* app., an effect further supported by fecal microbiota transplantation experiments (Wang L. et al., 2022).

The downregulation of antimicrobial peptides (AMPs) is an important factor contributing to the increased susceptibility of AD patients to microbial infections (Ong et al., 2002). In lesional skin, the expression of key AMPs, including human β-defensin 1, 2, and 3 (HBD-1, HBD-2, HBD-3), the cathelicidin LL-37 (Szabó et al., 2023), and its encoding gene cathelicidin antimicrobial peptide (CAMP) (Ma et al., 2022), is markedly reduced. This reduction compromises the skin’s innate immune defense capacity. Apigenin has been shown to robustly upregulate AMP expression in both *in vivo* and *in vitro* models. In a C57BL/6J mouse model of AD, apigenin significantly upregulated the expression of CAMP and mouse β-defensin 3 (mBD3), which may contribute to increased local antimicrobial activity (Hou et al., 2013). Similarly, in HaCaT model, apigenin was found to upregulate the expression of HBD-1, HBD-2, HBD-3, and LL-37, which may enhance antimicrobial peptide-mediated defenses under inflammatory conditions (Park C.-H. et al., 2020).

### 4.6 Modulation of programmed cell death

**FIGURE 6:**
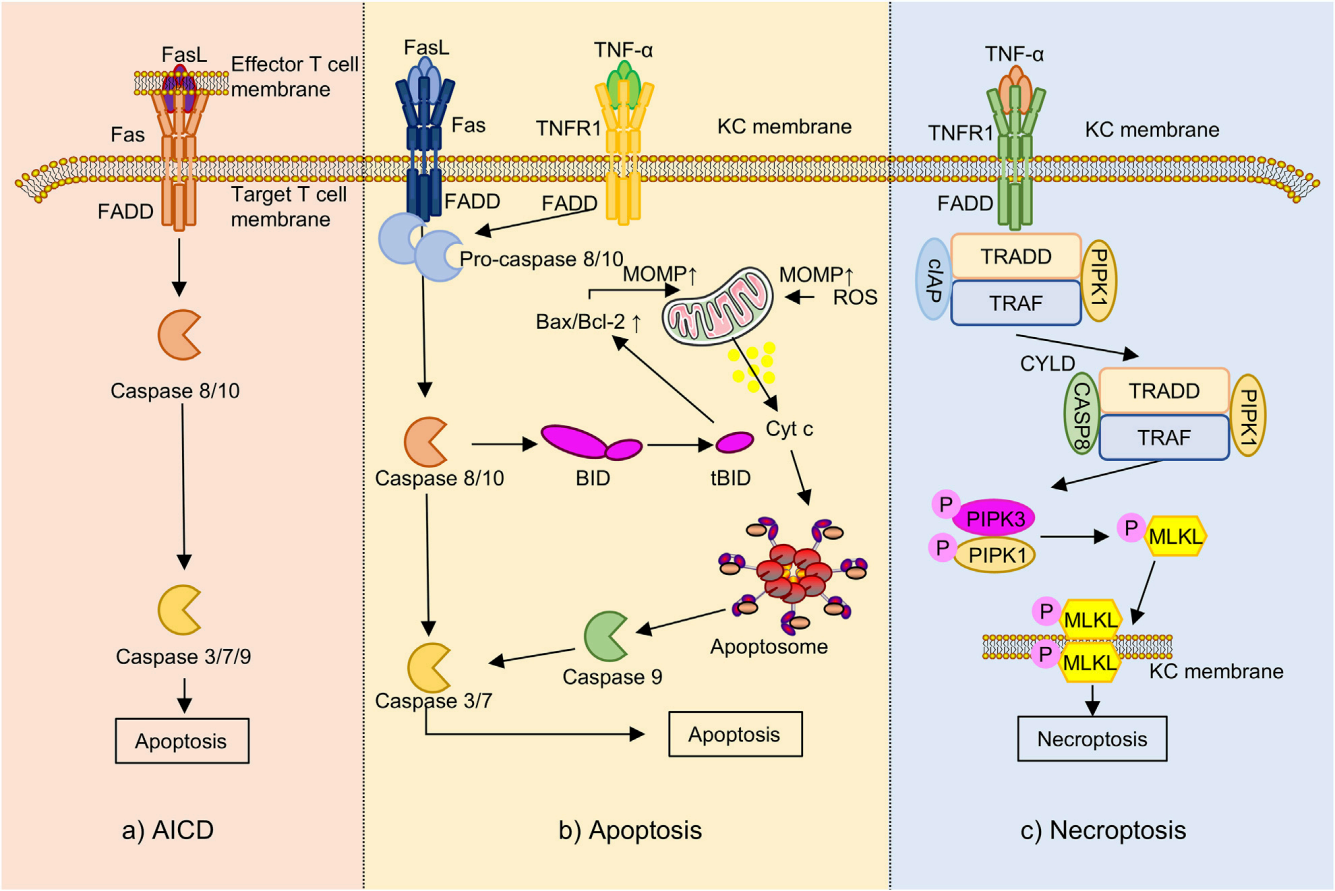
The programmed cell death underlying AD (the part that flavonoids can intervene in). Note: Fas, Fas cell surface death receptor; FasL, Fas ligand; FADD, Fas-associated death domain; TNFR1, tumor necrosis factor receptor 1; TRAF, TNF receptor-associated factor; cIAP, cellular inhibitor of apoptosis protein; KC, keratinocyte; BID, BH3-interacting domain death agonist; tBID, truncated BID; Bax, Bcl-2-associated X protein; Bcl-2, B-cell lymphoma 2; MOMP, mitochondrial outer membrane permeabilization; Cytc, cytochrome c; ROS, reactive oxygen species; RIPK-, receptor-interacting protein kinase; MLKL, mixed lineage kinase domain-like protein; TRADD, TNFR1-associated death domain protein; CYLD, cylindromatosis tumor suppressor; AICD, activation-induced cell death.

In AD pathogenesis, programmed cell death maintains immune homeostasis, modulates inflammation, and supports skin barrier integrity (Lossi, 2022; Tanaka et al., 2022). Beyond classical apoptosis, necroptosis (Luo C.-H. et al., 2024) and activation-induced cell death (AICD) (Cencioni et al., 2015) have also been implicated in AD progression and the regulation of immune and epithelial cell function (Figure 6).

Apoptosis is a caspase-dependent form of programmed cell death that proceeds through two major pathways: the extrinsic Fas cell surface death receptor/Fas ligand (Fas/FasL) axis and the intrinsic mitochondrial pathway (Rebane et al., 2012). In the extrinsic pathway, IFN-γ secreted by cutaneous lymphocyte-associated antigen-positive (CLA^+^) T cells upregulates Fas expression on keratinocytes (Trautmann et al., 2000), rendering them sensitive to FasL-mediated signaling. This cascade activates caspase-8 and downstream caspase-3 (Takahashi et al., 1999), leading to DNA fragmentation and keratinocyte apoptosis (Simon et al., 2006).

The intrinsic pathway is typically activated by TNF-α or oxidative stress, which disrupts the Bcl-2-associated X protein to B-cell lymphoma 2 (Bax/Bcl-2) ratio, promoting mitochondrial outer membrane permeabilization (MOMP) and cytochrome c release (Tait and Green, 2010), which subsequently activates caspase-9 and initiates the apoptotic cascade (Martinou and Youle, 2011). In AD, elevated expression of Fas and FasL increases keratinocyte vulnerability to IFN-γ-induced apoptosis (Rebane et al., 2012), thereby contributing to skin barrier breakdown and exacerbation of eczematous inflammation (Szymanski et al., 2018). In the TNF-α-stimulated HaCaT model, baicalin treatment was associated with attenuation of inflammatory injury via inhibition of the STAT3/NF-κB signaling pathways. This effect involved reduced production of pro-inflammatory cytokines and modulation of apoptotic regulators including Bax, Bcl-2, and caspase-3 (Wu et al., 2020).

Necroptosis is a regulated, caspase-independent form of programmed cell death mediated by the receptor-interacting protein kinase 1 (RIPK1), RIPK3, and mixed lineage kinase domain-like protein (MLKL) signaling axis (Fritsch et al., 2019). Although mechanistically distinct, it mimics necrosis morphologically, including membrane rupture and inflammatory content release (Karlowitz and van Wijk, 2023). Under caspase-8 inhibition, RIPK1/RIPK3 phosphorylation initiates necrosome formation and MLKL activation, triggering membrane breakdown and the release of pro-inflammatory damage-associated molecular patterns (DAMPs) (Fritsch et al., 2019; Meng Y. et al., 2021). In AD, TNF-α, oxidative stress, or microbial cues induce keratinocyte necroptosis, resulting in IL-33 release and ILC2 activation, thereby promoting Th2-skewed inflammation. This necroptotic axis is significantly elevated in AD lesions and correlates with disease severity, highlighting its role in chronic inflammation (Luo C.-H. et al., 2024). In a mouse model of AD, treatment with EGCG-loaded nanoparticles (EGCG-NPs) was associated with decreased expression of necroptosis-related markers including RIPK1, RIPK3, and MLKL, as well as a reduction in Terminal deoxynucleotidyl transferase dUTP Nick-End Labeling- (TUNEL-) positive keratinocytes, suggesting potential inhibition of keratinocyte necroptosis (Han et al., 2022). Additionally, the flavonoid nepetin has been reported to attenuate oxidative stress, suppress pro-inflammatory cytokines production, and reduce cell death by modulating the myeloid differentiation primary response 88 (MyD88) –mitogen-activated protein kinase kinase 3/6 (MKK3/6) –Akt signaling pathway, a potential role in the regulation of necroptosis or other non-apoptotic forms of programmed cell death (Gong et al., 2024).

AICD is a specialized form of programmed cell death that occurs in chronically stimulated T cells following prolonged antigen exposure, serving as a critical mechanism to maintain immune homeostasis and peripheral tolerance (Arakaki et al., 2014). AICD is primarily mediated through the Fas/FasL signaling axis. Upon sustained activation, T cells upregulate FasL, which binds to Fas receptors on themselves or neighboring cells, initiating caspase-8 activation and leading to apoptosis (Zhang et al., 2000; Nakano et al., 2007). This leads to the downstream activation of effector caspases, including caspase-3, -7, and -9, ultimately resulting in apoptotic cell death. Both *in vitro* and *ex vivo* studies have shown that Th1 cells exhibit greater susceptibility to AICD than Th2 cells, a difference largely attributed to more efficient Fas ligand surface expression in Th1 cells, which in turn contributes to the Th2-biased immune profile observed in AD (Oberg et al., 1997; Akdis et al., 2003). Recent research has reported that in DNCB and dust mite-induced AD mouse models, kaempferol attenuates disease symptoms, potentially through modulation of T cell overactivation and apoptosis. Mechanistic *in vitro* findings suggest that kaempferol preserves the expression of anti-apoptotic and apoptotic regulatory proteins, including Bcl-2 and caspase-3, -7, and -9, thereby reducing AICD-induced cell death in T cells (Lee and Jeong, 2021). Additionally, *in vivo* evidence indicates that kaempferol improves skin barrier function and attenuates oxidative stress (Nasanbat et al., 2023).

### 4.7 Attenuation of oxidative stress and redox imbalance

**FIGURE 7:**
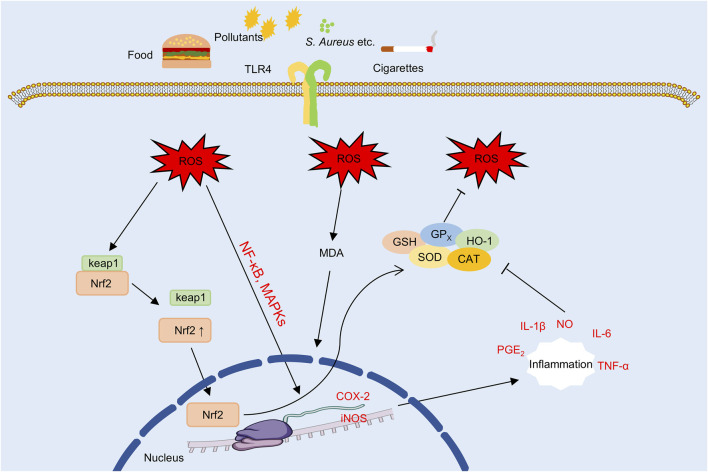
The oxidative stress underlying AD (the part that flavonoids can intervene in). Note: TLR4, Toll-like receptor 4; ROS, reactive oxygen species; MDA, malondialdehyde; NF-κB, nuclear factor kappa B; MAPK, mitogen-activated protein kinase; Keap1, Kelch-like ECH-associated protein 1; Nrf2, nuclear factor erythroid 2-related factor 2; GSH, glutathione; GP_X_, glutathione peroxidase; SOD, superoxide dismutase; CAT, catalase; HO-1, heme oxygenase-1; COX-2, cyclooxygenase-2; iNOS, inducible nitric oxide synthase; NO, nitric oxide; PGE_2_, prostaglandin E_2_.

Oxidative stress is increasingly recognized as a central pathogenic factor in the progression of AD (Galiniak et al., 2022), as shown in Figure 7. In lesional skin, excessive accumulation of ROS compromises the epidermal barrier, facilitates TEWL and allergen penetration, activates pro-inflammatory signaling cascades, and disrupts immune homeostasis (Raimondo et al., 2023). Together, these processes establish a self-perpetuating cycle of oxidative damage, inflammation, and immune dysregulation that contributes to the chronicity and severity of the disease.

Persistent accumulation of ROS induces lipid peroxidation in keratinocyte membranes and structural proteins, resulting in impaired barrier function and increased TEWL (Yang et al., 2022; Berdyshev, 2024). In parallel, ROS activate inflammatory signaling cascades such as the NF-κB and MAPK pathways, which promote the expression of pro-inflammatory cytokines including IL-1β, IL-6, and TNF-α, thereby reinforcing inflammation through a self-amplifying loop (Yang et al., 2022; Herath et al., 2024). Experimental evidence indicates that butein reduces ROS generation and suppresses IL-6 and intercellular adhesion molecule-1 (ICAM-1) expression in TNF-α-stimulated HaCaT model. These effects are associated with inhibition of MAPK and NF-κB pathway activation, suggesting potential antioxidant and skin barrier-protective properties activities *in vitro* (Seo et al., 2015). Similarly, in LPS-stimulated HaCaT model, nepetin has been shown to attenuate ROS accumulation and reduce the production of inflammatory cytokine, potentially through modulation of the MyD88-MKK3/6–Akt–NF-κB signaling pathway (Gong et al., 2024).

Downstream of ROS accumulation, COX-2 and iNOS are key pro-inflammatory enzymes that amplify oxidative and immune-mediated damage. COX-2 catalyzes the synthesis of prostaglandin E_2_ (PGE_2_) (Ilari et al., 2020), while iNOS produces nitric oxide (NO), which together contribute to the formation of reactive nitrogen species (RNS) (Zhao et al., 2022), thereby exacerbating tissue inflammation and oxidative injury. In addition, ROS promote Th2-skewed immune responses and impair Treg function (Nakajima et al., 2021), resulting in the upregulation of type 2 cytokines such as IL-4 and IL-13 (Fu Z. et al., 2024), which further exacerbate chronic allergic inflammation in AD. Among natural antioxidants, in LPS-stimulated RAW264.7 macrophages, apigenin has been shown to suppresses the expression of COX-2, iNOS, as well as the inhibition of the MAPK signaling pathway and in IgE-sensitized RBL-2H3 cells, it suppresses of the FcεRI signaling pathway, suggesting potential anti-allergic and anti-inflammatory activities (Park C.-H. et al., 2020). Similarly, quercetin and its derivatives have been shown to downregulate the expression of iNOS, COX-2, and Th2-associated cytokines (IL-4, IL-5, and TSLP), while reducing serum IgE levels and eosinophil counts in AD mouse model, suggesting that they may concurrently mitigate oxidative stress and modulate immune responses (Jafarinia et al., 2020).

To counteract the toxicity of ROS, the body depends on a complex network of endogenous antioxidant systems, including SOD, glutathione (GSH), glutathione peroxidase (GPx), and heme oxygenase-1 (HO-1) (Jegadheeshwari et al., 2024), which collectively maintain cellular redox homeostasis (Meca et al., 2021). Among these, SOD and GPx function cooperatively to neutralize ROS (Bhat et al., 2014; Ighodaro and Akinloye, 2018), GSH preserves the intracellular reducing environment, and HO-1 expression is upregulated by the nuclear factor erythroid 2-related factor 2 (Nrf2) (Liu F. et al., 2021; Peng et al., 2024), thereby enhancing cytoprotective responses. In contrast, malondialdehyde (MDA), a terminal product of lipid peroxidation, serves as a widely recognized biomarker of oxidative stress and cellular injury (Mas-Bargues et al., 2021). In DNCB-induced AD mouse model, EGCG-NPs were associated with increased activity of antioxidant enzymes such as SOD and GSH, decreased MDA levels, and enhanced total antioxidant capacity (T-AOC), collectively contributing to the restoration of epidermal redox balance (Han et al., 2022). In addition, quercetin has been reported to exert similar protective effects by activating the Nrf2/HO-1 signaling pathway, enhancing antioxidant enzyme expression, and mitigating hydrogen peroxide-induced oxidative stress in keratinocytes (Jafarinia et al., 2020).

## 5 Limitations and safety concerns in conventional oral and topical delivery of flavonoids

### 5.1 Pharmacokinetic limitations to the oral bioavailability of flavonoids

Flavonoids exhibit broad pharmacological activities, particularly in dermatological disorders. Nonetheless, their clinical translation is substantially limited by pharmacokinetic barriers. The low oral bioavailability of flavonoids results primarily from structural characteristics, transport mechanisms, metabolic clearance, and tissue distribution.

Most natural flavonoids exist predominantly as *O*-glycosides and *C*-glycosides, whose high hydrophilicity and limited lipophilicity restrict their ability to traverse the intestinal epithelium and enter systemic circulation. It is increasingly recognized that these glycosides are generally believed to require hydrolysis in the intestinal lumen by host enzymes or gut microbiota into more membrane-permeable aglycones for efficient absorption (Nakamura et al., 2020). Animal studies further confirm that unmetabolized glycosides, such as quercetin-3-*O*-sophoroside, cannot penetrate the intestinal epithelium (Nakamura et al., 2018). *O*-glycosides (e.g., isoquercitrin) are typically hydrolyzed in the upper gastrointestinal tract to release aglycones like quercetin, while *C*-glycosides (e.g., orientin, vitexin), due to their structural stability, are only metabolized into absorbable forms in the colon by gut microbiota (Xie et al., 2022). Notably, human gut microbiota can transform *C*-glycosides such as orientin and vitexin into their corresponding aglycones, luteolin and apigenin, suggesting an important role of the gut microbiota as a key contributor to their metabolic conversion (Wang S. et al., 2022).

Metagenomic analyses reveal that while *O*-glycosidases are widely expressed in the gut microbiota, their expression levels vary significantly among individuals, leading to highly personalized hydrolysis efficiency and absorption outcomes (Goris et al., 2021). This is supported by the observation that glycosylated flavonoids are nearly undetectable in plasma, with aglycones generally considered to be the predominant bioactive forms (Chen et al., 2022).

Flavonoid absorption is also influenced by transmembrane transport mechanisms. Studies using the Caco-2 Brush Border Expressing clone 1 cell line (Caco-2 BBe1) model demonstrate that glycosylated flavonoids are primarily absorbed via Sodium-dependent glucose transporter 1- and Glucose transporter 2-mediated active transport, whereas aglycones are mainly taken up by passive diffusion (Zhang et al., 2020). These mechanisms not only affect transepithelial flux but also play a key role in determining oral bioavailability.

Even after successful absorption into the circulatory system, flavonoids face significant first-pass metabolic barriers. In both the intestinal epithelium and liver, they are rapidly transformed into hydrophilic metabolites through phase II enzymatic reactions such as glucuronidation, sulfation, and methylation, catalyzed by enzymes including uridine 5′-diphospho-glucuronosyltransferase, sulfotransferase, and catechol-*O*-methyltransferase. These transformations are generally associated with reduced bioactivity and lower plasma concentrations (Najmanová et al., 2020). For instance, the oral bioavailability of morin is only 0.45%, whereas systemic exposure reaches 92.9% following intravenous administration, clearly illustrating the limiting impact of first-pass metabolism on the pharmacological potential of oral administration (Li et al., 2019).

In addition, flavonoids also undergo phase I metabolism mediated by hepatic cytochrome P450 (CYP450) enzymes. For example, EGCG can be oxidatively metabolized by CYP450 enzymes (Zhao et al., 2019), while quercetin undergoes glucuronidation by uridine 5′-diphospho-glucuronosyltransferase 1A1 to form quercetin-3-glucuronide, which can re-enter systemic circulation via enterohepatic recycling (Zhang et al., 2020). Although this recycling prolongs the residence time of flavonoids in the body, it is often accompanied by reduced bioactivity, posing challenges for maintaining consistent pharmacological activity.

Furthermore, flavonoids predominantly circulate in conjugated forms and exhibit high plasma protein binding affinity, which limits their distribution to target tissues. Although flavonoids are detectable in organs such as the liver, kidneys, and lungs, their concentrations at disease sites are typically low and transient, thereby limiting their therapeutic potential (Teng et al., 2023). Additionally, several flavonoids exhibit remarkably short plasma half-lives. For example, ikarisoside A exhibits a half-life of 3.15 h and a clearance rate of 42.9 L/h/kg (Cong et al., 2018), while isoformononetin has a half-life of just 1.9 h and an oral bioavailability of 21.6% (Raju et al., 2019). These pharmacokinetic features, characterized by rapid clearance and short systemic exposure, further challenge their preclinical-to-clinical applicability.

### 5.2 Limitations of conventional topical flavonoid delivery

Although certain flavonoids such as naringenin and flavanone exhibit enhanced skin permeability under inflamed or barrier-compromised conditions, their transdermal absorption remains limited in intact skin, often necessitating the use of penetration enhancers to achieve therapeutic depth (Alalaiwe et al., 2020). The stratum corneum, with its tightly packed lipid matrix, serves as a major barrier impeding the cutaneous deposition of natural bioactive metabolites including flavonoids (Wang et al., 2024).

Moreover, key flavonoids such as quercetin (Khursheed et al., 2020) and fisetin (Szymczak and Cielecka-Piontek, 2023) exhibit extremely poor aqueous solubility and high lipophilicity, resulting in limited dispersibility in topical formulations and thereby potentially limiting skin permeation. As a result, their unformulated free forms demonstrate minimal cutaneous bioavailability and markedly limited topical pharmacological potential.

In addition, flavonoid molecules generally exhibit poor physicochemical stability under conditions such as light exposure, elevated temperature, and pH fluctuations, making them prone to degradation and potentially affecting their pharmacological activity. In particular, the phenolic hydroxyl groups are highly reactive and susceptible to oxidation, cleavage, and photochemical transformation. A spectroscopic analysis involving 177 flavonoid metabolites revealed their pronounced instability under ultraviolet irradiation, with this issue being particularly relevant in topical application settings (Taniguchi et al., 2023). For example, free quercetin displays structural instability under light or oxidative stress, resulting in a substantial decline in both antioxidant and anti-inflammatory activities, potentially rendering it therapeutically ineffective (Vale et al., 2021).

### 5.3 Potential toxicities and safety considerations of oral flavonoids

While flavonoids are valued for their therapeutic effects, accumulating evidence indicates that high doses or prolonged exposure can result in toxicity, highlighting the need for a comprehensive safety assessment in future research.

With respect to hepatotoxicity, animal studies have demonstrated that high doses of quercetin can markedly elevate serum transaminase levels and induce lipid peroxidation as well as glutathione depletion in hepatic tissues (Singh et al., 2021), indicating that excessive quercetin intake may contribute to hepatocellular injury by activating oxidative stress pathways. Similarly, under oxidative stress, EGCG not only failed to provide protection in hydrogen peroxide-induced model but also worsened mitochondrial membrane potential loss, caspase-3 activation, and DNA fragmentation, suggesting potential pro-apoptotic and mitochondria-damaging effects under such conditions (Sahadevan et al., 2023).

In terms of genotoxicity, isoquercitrin, a quercetin derivative, has demonstrated mild mutagenic activity in the Ames test, indicating that potential DNA damage may occur under certain structural or metabolic conditions (Kapoor et al., 2022). Regarding endocrine disruption, common flavonoids such as genistein, quercetin, and apigenin have been reported to activate estrogen receptor signaling pathways at low concentrations, thereby exhibiting phytoestrogen-like activity (Zhang and Wu, 2022). These findings indicate that, at certain exposure levels, these metabolites have the potential to disrupt endocrine homeostasis and increase the risk of reproductive or developmental toxicity.

*In vitro* studies have shown that certain flavonoids, at high concentrations, can inhibit the growth of probiotic bacteria such as *Lactobacillus* app., while promoting the proliferation of opportunistic pathogens and inducing the accumulation of harmful metabolites, including H_2_S and NH_3_ (Pan et al., 2023), which may adversely affect gut microbial homeostasis under specific conditions.

Moreover, quercetin and its metabolites have been shown *in vitro* to exert weak to moderate inhibitory effects on key drug-metabolizing enzymes, including cytochrome P450 isoforms CYP3A4 and CYP2C19, as well as several major drug transporters (Mohos et al., 2020). These findings raise concerns that high-dose quercetin supplementation could potentially interfere with the metabolism and clearance of co-administered drugs, thereby raising the possibility of clinically relevant drug-drug interactions.

Given the documented risks associated with high-dose or long-term use of flavonoids, including hepatotoxicity, genotoxicity, endocrine disruption, gut microbiota dysbiosis, and drug interactions, it is imperative to develop optimized oral and topical delivery strategies. Rational modulation of dosage and release kinetics is equally essential to facilitate the clinical translation and standardized application of flavonoid-based therapies.

## 6 Delivery of flavonoids using novel strategies

### 6.1 Topical delivery strategies for flavonoids

Innovative transdermal delivery systems have demonstrated measurable improvements in enhancing the topical bioavailability of poorly soluble agents. For example, a micelle-in-hydrogel formulation containing only 0.075% hydrocortisone achieved a 9.2-fold increase in skin flux and a 50-fold improvement in cumulative permeation compared to a 1% commercial cream, revealing the advantage of follicular targeting in transdermal drug delivery (Yuan et al., 2020). Similarly, dissolvable microneedles loaded with 0.25% dexamethasone enabled rapid intradermal release and effectively attenuated AD symptoms, reducing epidermal thickness by >70% and spleen index by over 50%, without observable toxicity (Ben David et al., 2023). Moreover, accumulating evidence supports the potential of novel transdermal systems to enhance flavonoid delivery and therapeutic effects in preclinical AD models (Table 2).

**TABLE 2:** Topical delivery strategies for flavonoids in AD.

Delivery system	Flavonoid(s)	Carrier materials	Key results	References
Microneedles	EGCG + Ascorbic acid	Poly (γ-glutamic acid), dissolvable microneedle	95% EGCG retention after 4 weeks; skin delivery up to 600 μm; serum IgE reduced from 12156 to 5555 ng/mL	Chiu et al. (2021)
Polymeric nanoparticles	EGCG	PEG-PLGA nanoparticles	Reduced epidermal thickness from 109.4 μm to 43.6 μm; improved antioxidant enzymes (SOD, GSH, T-AOC) by 35%–60%	Han et al. (2022)
Hydrogel	Naringenin	Carboxymethyl cellulose/2-hydroxyethyl acrylate hydrogel	Enhanced release at pH 8.5 (73%) vs. pH 5.5 (42%); 58.8% skin permeation; >90% HaCaT cell viability	Park et al. (2018)
Microsponge	Naringenin	Ethyl cellulose-based microsponge, Carbopol gel	92.3% cumulative release; skin deposition 802.9 μg/cm^2^; reduced ear thickness and WBC count *in vivo*	Nagula and Wairkar (2020)
Liposome	Taxifolin	Liposome	Clinical score reduction: 72.3% (TAX) vs. 82.2% (TAX + L); greater IgE suppression and hydration restoration with TAX + L	Kim et al. (2015)

Note: EGCG, epigallocatechin-3-gallate; IgE, immunoglobulin E; PEG, polyethylene glycol; PLGA, poly (lactic-co-glycolic acid); SOD, superoxide dismutase; GSH, glutathione; T-AOC, total antioxidant capacity; WBC, white blood cells; TAX, taxifolin; TAX + L, taxifolin + liposome.

#### 6.1.1 Microneedles

A promising approach to enhance the topical delivery of flavonoids is the use of microneedle systems, which painlessly penetrate the stratum corneum and enable direct intradermal deposition of active metabolites (Luo Z. W. et al., 2024). For example, dissolvable poly (γ-glutamic acid) microneedles co-loaded with EGCG and L-ascorbic acid retained 95% EGCG and 93% antioxidant activity after 4 weeks at 4 °C, and delivered 63% EGCG and 62% ascorbic acid up to 600 μm into the skin. A once-weekly dose (358 μg EGCG, 438 μg ascorbic acid) reduced serum IgE from 12,156 to 5,555 ng/mL and histamine from 81 to 40 pg/mL, highlighting their low-dose, high-efficiency therapeutic potential (Chiu et al., 2021).

#### 6.1.2 Polymeric nanoparticles

Another widely explored approach for enhancing the delivery efficiency of flavonoids is nanoscale encapsulation, which enhances solubility, physicochemical stability, and skin-targeting efficiency (Huang P.-H. et al., 2016). A recent study showed polyethylene glycol-poly (lactic-co-glycolic acid) (PEG–PLGA) nanoencapsulation of EGCG was shown to reduce epidermal thickness from 109.4 μm to 43.6 μm after 21 days of topical application, accompanied by decreases in lactate dehydrogenase and MDA, and increases SOD, GSH, and T-AOC by 35%–60%. Compared to free EGCG, the nanoparticles showed greater suppression of IL-4, TNF-α, and IL-17A, which may contribute to modulating Th1/Th2/Th17 immune balance (Han et al., 2022).

#### 6.1.3 Hydrogels

As semi-solid matrices with excellent biocompatibility and hydration properties, hydrogel-based systems have been proposed as a favorable platform for controlled flavonoid delivery and enhanced dermal deposition (Arora and Nanda, 2019). Notably, a pH-responsive carboxymethyl cellulose/2-hydroxyethyl acrylate hydrogel was reported to enhance the stability and transdermal delivery of naringenin. After 30 days at 40 °C and 75% humidity, the formulation maintained stable release. Drug release reached 73% at pH 8.5, compared to 42% at pH 5.5. Skin permeation efficiency increased to 58.8%, significantly outperforming conventional 1,3-butylene glycol systems (43.5% and 42.4%), with reduced stratum corneum retention and improved dermal deposition. No cytotoxicity was observed in HaCaT cells, with cell viability maintained above 90%, and the system achieved high dermal delivery efficiency at a moderate drug loading level of 51.5% (Park et al., 2018).

#### 6.1.4 Microsponges

Microsponge-based carriers have been investigated as a potential platform for transdermal flavonoid delivery, providing a porous structure conducive to prolonged release and enhanced skin deposition (Grillo et al., 2019). Ethyl cellulose-based microsponges loaded with naringenin achieved a cumulative *in vitro* release of 92.3% within 24 h. Compared to the plain gel, the Carbopol gel formulation (NGMSG1%) exhibited a 2.1-fold release (87.5% vs. 42.4%) and achieved a skin deposition of 802.9 μg/cm^2^, approximately 3.7 times greater than that of the conventional formulation. *In vivo*, the formulation significantly reduced ear thickness and white blood cell counts, indicating sustained-release performance, improved skin retention, and notable anti-inflammatory effects (Nagula and Wairkar, 2020).

#### 6.1.5 Liposomes

In addition, liposomal carriers have been shown to enhance transdermal absorption and immunomodulatory effects of flavonoids (Zhang and Michniak-Kohn, 2020). Topical application of 1% taxifolin (TAX) glycoside significantly attenuated TNCB-induced AD-like lesions, with clinical scores reduced by 72.3% and 82.2% in the TAX and TAX + L (liposome) groups, respectively. TAX treatment decreased TEWL and increased skin hydration, while TAX + L restored hydration to normal levels, with a strong inverse correlation between TEWL and hydration (r = −0.61). Both treatments markedly reduced serum IgE levels, with greater suppression observed in the TAX + L group, accompanied by downregulation of IL-4 and upregulation of IFN-γ. Liposomal encapsulation significantly enhanced transdermal delivery and barrier recovery, suggesting potential benefits in terms of stability, permeability, and immune modulation (Kim et al., 2015).

Taken together, these innovative delivery platforms have been shown to enhance the transdermal bioavailability of flavonoids and improve their pharmacological effects and biocompatibility. Such multi-dimensional advantages may support the rational design of flavonoid-based topical formulations with potential for clinical translation in AD and related inflammatory skin disorders.

### 6.2 Oral delivery strategies for flavonoids

Despite their diverse pharmacological activities, the systemic application of flavonoids remains constrained by limited aqueous solubility, low oral bioavailability, rapid metabolic clearance, and insufficient targeting to skin and immune tissues. While direct evidence in AD models remains scarce, oral delivery systems developed in oncology and inflammatory conditions may provide mechanistic insights with potential translational relevance (Vazhappilly et al., 2021).

For example, poly (lactic-co-glycolic acid) nanoparticles co-loaded with baicalin and CpG oligodeoxynucleotides promoted macrophage repolarization and T cell-mediated cytotoxicity in tumors (Han et al., 2021), whereas dual-drug nanostructured lipid carriers functionalized with R9dGR peptide achieved >73% tumor inhibition without systemic toxicity (Ma et al., 2020). In inflammatory models, cerium oxide–quercetin nanocomposites (CeO_2_@QU) effectively scavenged ROS and contributed to the maintenance of redox homeostasis (Wang et al., 2021). Exosome-based systems further expand this potential, providing biocompatible and targetable platforms for oral flavonoid delivery (Ferreira et al., 2022). Notably, hesperidin-loaded bovine milk exosomes enhanced oral bioavailability by approximately 2.5-fold and significantly suppressed melanoma progression *in vivo* with no apparent systemic toxicity (Kumar et al., 2024).

Taken together, these findings suggest a mechanistic basis for exploring for oral flavonoid delivery in AD, although targeted preclinical validation remains essential.

### 6.3 Limitations of novel delivery systems

Although recent studies have reported substantial improvements in the delivery efficiency and therapeutic performance of flavonoids using advanced delivery systems, including enhanced stability, bioavailability, and targeting efficiency (Costa et al., 2021; Delgado-Pujol et al., 2025), several practical and clinical limitations have been identified, which merit further investigation and refinement.

For topical administration routes, including dermal and ocular delivery, hydrogels and nanogels are often limited by suboptimal skin permeability, poor adhesiveness, and challenges in large-scale manufacturing (Grillo et al., 2019; Labie and Blanzat, 2023). While microneedle technology effectively overcomes the stratum corneum barrier, its clinical application remains constrained by low drug-loading capacity and insufficient mechanical strength (Han et al., 2025). Liposomal formulations, commonly used for local and ocular delivery, may still be affected by particle size heterogeneity, suboptimal encapsulation efficiency, and difficulties in industrial-scale production (Akar et al., 2024). Even with chitosan surface modification, such systems have shown limitations in overcoming mucosal barriers, which may reduce their retention and permeability (Khalil et al., 2020). Moreover, targeted microsponge-based gels developed for dermal diseases have exhibited variable pharmacodynamic effects and inconsistent release profiles (Nagula and Wairkar, 2020).

For oral administration, polymeric nanoparticles offer controlled release and high drug-loading potential, but are prone to protein corona formation *in vivo*, which compromises targeting capabilities. In addition, degradation within the gastrointestinal tract and first-pass metabolism substantially reduce systemic bioavailability, and preclinical animal findings may not consistently predict human pharmacokinetics and efficacy (Beach et al., 2024). Although hydrogels and nanogels hold promise in oral formulations, their physicochemical stability and delivery accuracy under dynamic digestive conditions remain suboptimal (Delgado-Pujol et al., 2025). Therefore, despite notable advances in enhancing flavonoid pharmacokinetics, both topical and oral delivery systems still require further optimization to meet clinical standards. In particular, greater emphasis is required on material design, biological barrier modulation, scalable fabrication, and *in vivo* transformation mechanisms to meet the stringent demands for safety, stability, reproducibility, and standardization necessary for clinical translation.

## 7 Clinical experimental research

Recent clinical investigations have begun to explore the therapeutic potential of natural flavonoids in AD, with early-phase studies suggesting possible reductions in inflammation, pruritus, and skin barrier impairment. Notably, flavonoids such as quercetin, silymarin, EGCG, and grape seed proanthocyanidins have shown reported to exhibit beneficial effects in topical and systemic interventions, with preliminary data indicating acceptable safety profiles (Table 3).

**TABLE 3:** Clinical experimental research.

Metabolites/Drugs	Population	Design	Intervention	Outcomes	References
Silymarin + *Fumaria officinalis* L. extract	40 eczema patients	Randomized, double-blind controlled clinical trial	Topical cream with *Fumaria officinalis* L. extract and silymarin vs. mometasone 0.1%	SCORAD score reduced from 26.05 ± 7.1 to 6.94 ± 2.6 in botanical drug group (p = 0.04); comparable to mometasone group (27.66 ± 5.9 to 4.77 ± 1.6, p = 0.03); no adverse effects reported	Iraji et al. (2022)
Quercetin	30 healthy volunteers; UV, histamine, SLS, GA-induced skin stress	Single-blind clinical study	Topical Quercevita^®^ (1% quercetin phytosome)	Quercetin 1% significantly reduced erythema (−10.05%, p = 0.00329), wheal diameter (−13.25%), and itch VAS score after histamine stimulation; improved hydration and reduced TEWL vs. placebo	Maramaldi et al. (2016)
Silymarin	Number of subjects not specified; topical application in AD	Formulation development and clinical application	Silymarin-loaded (PLO)	Optimized PLO (20% pluronic, 3% lecithin) showed enhanced silymarin skin penetration; *In vivo* topical application significantly reduced erythema, swelling, and inflammation in AD patients	Mady et al. (2016)
EGCG + Vitamin E + Grape seed procyanidins	44 patients with mild-to-moderate AD (face/neck)	Randomized, controlled, double-blind clinical study	MD2011001 cream (vitamin E, EGCG, grape seed procyanidins)	MD2011001 reduced Investigator’s Global Assessment (IGA) score significantly over 28 days; faster lesion area reduction vs. placebo; well tolerated on face and periocular regions (n = 44)	Patrizi et al. (2016)
Whey protein + Dodder (*Cuscuta campestris* Yunck.) seed extract	52 adults with moderate-to-severe AD	Randomized, double-blind, placebo-controlled clinical trial	Oral whey protein with *Cuscuta campestris* Yunck. extract	After 15-day treatment, skin moisture and elasticity significantly increased (p < 0.001); pruritus and sleep disturbance significantly reduced at day 15 (p < 0.05) and day 30 (p < 0.001); pigmentation also decreased (p < 0.001); no serious adverse effects	Mehrbani et al. (2015)

Note: EGCG, epigallocatechin-3-gallate; GA, glycolic acid; PLO, pluronic-lecithin organogel; SCORAD, SCORing, Atopic Dermatitis; SLS, sodium lauryl sulfate; UV, ultraviolet radiation; VAS, visual analog scale; TEWL, transepidermal water loss.

As a classical flavonoid complex, silymarin presents notable challenges in transdermal delivery, which have prompted formulation innovations. A preclinical study developed a Pluronic lecithin organogel (PLO) system to enhance the dermal delivery of silymarin. Topical application in AD patients resulted in visible reductions in erythema and inflammation (Mady et al., 2016). Although the study lacked a control group and primarily focused on formulation optimization and percutaneous absorption, the observed outcomes may support the need for further clinical investigation. A randomized, double-blind, controlled clinical trial evaluated the clinical therapeutic efficacy of a topical botanical drug cream containing *Fumaria officinalis* L. extract and silymarin in patients with mild to moderate AD (Iraji et al., 2022). A total of 40 patients were enrolled and randomly assigned to receive either the botanical drug cream or 0.1% mometasone cream for 2 weeks. Both groups exhibited significant reductions in SCORing Atopic Dermatitis (SCORAD) scores, with no significant difference between them, indicating that the botanical drug formulation was non-inferior to low-potency corticosteroid treatment. These findings support the anti-inflammatory potential of silymarin and its synergistic plant-derived metabolites in clinical settings.

In another single-blind human study using a skin irritation model, the soothing and antipruritic effects of 1% Quercevita^®^ cream, a phytosomal formulation of quercetin, were assessed (Maramaldi et al., 2016). Thirty healthy volunteers were subjected to induced skin irritation via UV exposure, histamine, sodium lauryl sulfate, and fruit acids. The quercetin formulation significantly reduced erythema, wheal formation, and subjective itch scores, exhibiting anti-inflammatory effects comparable to 1% desloratadine cream and supporting its potential utility as a topical anti-inflammatory agent. A double-blind randomized controlled trial also investigated the therapeutic efficacy and safety of MD2011001, a non-steroidal combination cream containing EGCG, grape seed proanthocyanidins, and vitamin E, in 44 patients with mild to moderate AD involving the face and neck (Patrizi et al., 2016). After 28 days of treatment, significant reductions in lesion size and improvements in Investigator Global Assessment (IGA) scores were observed. The formulation was well tolerated, with no serious adverse events reported.

In addition to topical applications, the oral administration of flavonoids has also received preliminary clinical investigation. A randomized, double-blind, placebo-controlled clinical trial involving 52 adults with moderate to severe AD assessed the efficacy of oral supplementation with whey protein combined with *Cuscuta campestris* Yunck. seed extract (Mehrbani et al., 2015). This combination contains multiple flavonoids including quercetin, kaempferol, and rutin. After 15 days of administration, patients exhibited notable improvements in skin hydration, elasticity, pruritus, and sleep disturbances. These improvements persisted for at least 2 weeks after treatment cessation, supporting a sustained symptomatic relief and skin barrier-restorative effects.

Collectively, current clinical studies suggest that natural flavonoids, particularly quercetin, silymarin, EGCG, and grape seed proanthocyanidins, may exert therapeutic effects in both topical and systemic interventions for AD, with generally favorable safety profiles. However, most trials to date have involved small sample sizes, lacked placebo controls, or utilized multi-metabolite formulations, limiting the interpretability of individual flavonoid effects. Future studies should prioritize large-scale, well-controlled clinical trials with optimized dosage forms and advanced delivery systems to validate efficacy, assess safety, and define appropriate treatment strategies.

## 8 Discussion and perspectives

AD is a chronic multifactorial inflammatory skin disease resulting from the interplay of genetic, immunological, environmental, and microbial factors. Although corticosteroids and biologics have shown clinical benefits, their long-term use is often constrained by high costs, adverse effects, heterogeneous patient responses, and limited applicability across AD subtypes. Consequently, these limitations have catalyzed increasing interest in natural metabolites with multi-target pharmacological properties and favorable safety profiles.

As highlighted in this review, flavonoids have emerged as promising therapeutic candidates due to their broad-spectrum actions on key pathophysiological processes in AD, including immune modulation, oxidative stress attenuation, barrier restoration, microbial regulation, pruritus relief, and regulation of programmed cell death. In particular, we extend current understanding by incorporating underexplored yet mechanistically relevant dimensions, such as the skin–gut axis, necroptosis, and AICD, thus providing a more integrated and updated perspective compared with existing literature.

However, despite robust pharmacodynamic potential in preclinical studies, the clinical translation of flavonoids remains hindered by multiple challenges, including poor solubility, low oral and dermal bioavailability, rapid systemic clearance, and insufficient toxicological profiling in humans. Moreover, the lack of standardized formulations, dose–response analyses, and long-term safety data significantly impedes regulatory progress and scientific reproducibility.

Although flavonoids are widely explored as multi-target natural metabolites in the pharmacological interventions for AD, some structurally reactive metabolites may function as pan-assay interference compounds (PAINS), thereby confounding mechanistic interpretation. PAINS often cause false-positive results through nonspecific redox activity, metal chelation, covalent protein modification, or membrane disruption (Baell, 2016; Bolz et al., 2021). For example, EGCG and quercetin can oxidize into ortho-quinones that non-selectively react with nucleophilic residues, leading to broad, non-targeted activity (Baell, 2016). Other flavonoids without classical redox structures, such as genistein and resveratrol, have also been shown to disturb membrane dipole potential or lower energy barriers, which can alter protein conformation and interfere with signaling (de Matos et al., 2021; Magalhães et al., 2022). Without orthogonal or *in vivo* validation, such effects may mislead interpretation, especially in *in vitro* models evaluating membrane-related functions (Bolz et al., 2021). Given that many mechanistic insights presented in this review are primarily derived from *in vitro* studies, and rigorously designed *in vivo* studies remain limited, future research should aim to reduce potential PAINS-related artifacts and enhance translational relevance by integrating pharmacokinetic and pharmacodynamic data, systematically evaluating membrane-related interference, and rigorously validating target specificity.

Furthermore, to address translational bottlenecks, we propose three strategic directions for future research. First, advanced delivery platforms, including microneedles, nanoparticles, and hydrogel-based systems, should be designed to enhance cutaneous retention, epithelial penetration, and pharmacokinetic stability. Concurrently, oral bioavailability can be improved through structural optimization, bioenhancer co-administration, or targeted nanoformulations that increase gastrointestinal absorption and metabolic resistance. Second, well-powered and mechanistically informed clinical trials are required to validate therapeutic efficacy, define human dosing thresholds, and ensure long-term safety. Third, the implementation of standardized manufacturing and quality control systems will be critical to ensure consistency, scalability, and regulatory compliance. Together, these approaches will be instrumental in transforming flavonoids from preclinical candidates to standardized therapeutic options for AD, while minimizing confounding assay artifacts and enhancing translational relevance.

## 9 Conclusion

In summary, flavonoids constitute a promising multi-target therapeutic platform for AD, providing a robust mechanistic foundation for translational advancement. Addressing pharmacokinetic limitations and safety concerns, alongside the advances in delivery strategies, could support the development of flavonoid-based therapies as disease-modifying interventions. These efforts align with precision dermatology’s emphasis on targeted, mechanism-informed care that integrates epithelial, immune, and microbial dimensions. Realizing this potential will require sustained collaboration across dermatology, pharmacology, toxicology, and pharmaceutical science to ensure the safe, efficacious, and scalable clinical application of flavonoid-based interventions.
